# Implementation and effects of social protection programs for children, older adults, and people with disabilities in Brazil and Ecuador: A scoping review

**DOI:** 10.1371/journal.pgph.0005281

**Published:** 2025-10-29

**Authors:** Julia M. Pescarini, Ana L. Moncayo, Joanna M N. Guimarães, Francine S. Dias, Ronald Ruiz, Samuel A G. da Silva, Gustavo Casais, Michal Shimonovich, Valerie Wells, Mhairi Campbell, Mauricio L. Barreto, S Vittal Katikireddi, Gustavo Matta, Peter Craig, on behalf of the Global Health Research Unit on the Social and, Environmental Determinants of Health Inequalities (SEDHI)

**Affiliations:** 1 Department of Infectious Disease Epidemiology & International Health, Faculty of Epidemiology and Population Health, London School of Hygiene & Tropical Medicine, London, United Kingdom; 2 Centro de Integração de Dados e Conhecimentos para Saúde (Cidacs), Instituto Gonçalo Moniz, Fundacao Oswaldo Cruz, Salvador, Brazil; 3 Centro de Investigación para la Salud en América Latina (CISeAL), Pontificia Universidad Católica del Ecuador, Quito, Ecuador; 4 Universidad Internacional del Ecuador, Quito, Ecuador; 5 Faculdade de Economía, Universidade Federal da Bahia, Salvador, Brazil; 6 MRC/CSO Social & Public Health Sciences Unit, University of Glasgow, Glasgow, United Kingdom; Chulalongkorn University College of Public Health Sciences, THAILAND

## Abstract

We conducted a scoping review to investigate planned (intentional) and unplanned (not intentional) effects of social protection on socioeconomic determinants of health (SDH) and health inequalities among children, adolescents, elders and people with disabilities, and their caregivers. We reviewed seven programs in (i) Brazil (*Programa Bolsa Familia* (BFP) and *Beneficio de Prestacao Continuada* (BPC)), and (ii) Ecuador (*Bono de Desarrolo Humano (BDH)*, *Bono 1000 días*, *Pensión Mis Mejores Años/Pensión para Adultos Mayores*, *Pensión Toda una Vida/Pensión para personas con discapacidad* and *Bono Joaquín Gallegos Lara)*. We searched PubMed, EMBASE, LILACS, Scopus, Econlit, PsycINFO, Global Health, Global Index Medicus and grey literature for studies evaluating program implementation and effects on health outcomes or SDH from 1990 to 2023. We extracted data from 114 studies (84 on BFP, 17 on BDH and 13 on BPC). No studies were identified for the remaining programs. In Brazil, we found substantial evidence of BFP planned effects on children’s health and some SDH but little evidence on its unplanned effects in adults and caregivers. Evidence effects of BPC on health outcomes were scarce, with only one study in elders and none among people with disabilities. In Ecuador, we found evidence only for BDH, with some studies on planned health effects and few on SDH and its unplanned effects. Very few studies used longitudinal data, quasi-experimental designs, or comparison groups of eligible non-recipients. Finally, we found large variations coverage and implementation of programs. In summary, our review highlights the lack of evidence on the overall impacts of social protection in Ecuador, particularly those targeting older adults and people with disabilities. In Brazil, further research is needed on unplanned health effects of the BFP and on the impacts of social protection targeting elders and people with disabilities.

## Introduction

There is a well-established multidimensional association between poverty and poor health outcomes [1,2]. Poverty is significant in the context of Latin America (LA) due to its history of social and political crisis and high levels of income inequality. Historical social inequalities, with a substantial number of children and older adults experiencing poverty, pushed many LA governments to invest, during the 1990s, in compensatory or pilot initiatives to address these issues [3]. These early programs laid the groundwork for later reforms, especially under progressive governments in the 2000s, that consolidated cash transfer mechanisms into broader social protection strategies aimed at alleviating poverty and improving quality of life [4,5]. Programs or policies included non-contributory pension schemes (also referred to in some contexts as “social pensions”, social allowances or, in some places, ‘bonos’) for elders or people with disabilities experiencing poverty as well as cash transfer programs targeting families facing poverty [4,6].

Conditional cash transfers such as *Prospera/Oportunidades* in Mexico, *Programa Bolsa Familia* in Brazil, *Bono de Desarollo Humano* in Ecuador, and *Juntos* in Peru, have shown significant impacts in reducing poverty and inequalities, improving school attendance and narrowing educational gaps, fostering economic inclusion, and directly (i.e., through cash and/or conditionalities) or indirectly improving some health outcomes [7–10]. However, conditional cash transfers focus on early life while, by 2020, over 30% of the LA population was living below the poverty line, over 50% of the population aged 15 or older was employed in the informal sector (i.e., defined as a low-productivity), and only 46.4% were contributing to pension systems [11].

In the recent context of economic crises and political instability, with COVID-19 pandemic exacerbating levels of poverty and inequality in Latin America [12], changes that seek the efficacy or effectiveness of those programs. This scoping review draws on social protection data from Brazil and Ecuador, two Upper-middle income countries that have invested, implemented or improved the targeting of specific social protection programs focusing on families experiencing poverty but have not yet achieved universal and comprehensive models of more developed countries in LA [13]. The similarity of social protection schemes in both countries, especially those that focus on the well-being of children from families experiencing poverty and older people or people with disabilities experiencing poverty, permits a fair comparison of how countries implemented and evaluated the effects of these programs. To understand the effects of these programs and suggest areas where further research could inform changes and improvements to maximise the effects of current and future social protection programs [5], we mapped and summarised the available evidence on programs implementation and its effects on health outcomes and their socioeconomic determinants in the two countries.

## Methods

The scoping review protocol was registered at the Open Science Framework (OSF) platform (https://archive.org/details/osf-registrations-zw9e5-v1). The study is reported using PRISMA and other guidelines adapted to scoping reviews [14–16].

### Population, interventions, implementation and effects (PIIE)

*Population:* We analysed the body of literature published on the implementation and effects of programs targeting children, adolescents, elders, and people with disabilities in Brazil and Ecuador. Therefore, we have included studies looking either at the programs targeted population, i.e., children and adolescents (aged <18 years), elders (aged ≥60 years) and people with disabilities experiencing poverty; as well as their caregivers.

*Interventions*: We studied seven social protection programs. In Brazil, we studied the *Programa Bolsa Familia* (BFP) (Bolsa Familia Program) and the *Beneficio de Prestacao Continuada* (BPC) (Continuous Cash Benefit). In Ecuador, we studied the *Bono de Desarrolo Humano* (Human Development Grant), *Bono 1000 días* (First 1000 days Grant), *Pensión Mis Mejores Años/Pensión para adultos mayores* (Pension for Elderly), *Pensión Toda una Vida/Pensión para personas con discapacidad* (Pension for People with disabilities) and *Bono Joaquín Gallegos Lara* (Joaquín Gallegos Lara Allowance).

*Implementation:* For implementation studies, we included all research examining changes, challenges, and perceptions in implementing programs or policies at a single point in time, over time, or in specific settings. We considered measures of quality, speed, coverage, and control mechanisms across different years and contexts (e.g., levels of poverty or development, political circumstances, management levels).

*Effects:* For evaluation studies on the effects of social protection programs, we included outcomes that directly or indirectly affect the lives of beneficiaries and their caregivers, encompassing both socioeconomic determinants of health and a range of health outcomes. Eligible studies included: (i) qualitative research with a structured methodological framework, or (ii) quantitative evaluative studies with a comparison group of individuals, families, or aggregates (e.g., schools, municipalities) who either did not receive the benefit, received a different social protection benefit, or were assessed before and after receiving the benefit.

Detailed information is shown in S1 Table.

### Inclusion and exclusion criteria

We included (i) original studies or systematic reviews, (ii) published from 1^st^ January 1990–21st November 2024, (iii) written in Portuguese, Spanish, or English, and that were either (iv) quantitative or qualitative evaluations of the implementation of the seven selected social protection programs within Brazil or Ecuador, as well as comparisons between them at national and subnational levels; or (v) individual or aggregated studies that have evaluated the effects of the programs on the selected outcomes at national and subnational levels.

We excluded papers that did not match our aims in terms of (i) study design: simulation studies, commentaries, literature reviews or scoping reviews, (ii) study programs: studies comparing any of the seven selected programs with others not included in this scoping review, (iii) study population: studies that do not refer to evaluations performed specifically or partially on children or adolescents, elders and people with disabilities as well as on their caregivers, and (iv) comparison groups: effect evaluation studies without a counterfactual group (i.e., a comparison group that did not receive the intervention, that received a different amount of the intervention or a control period) or that included only a description of beneficiaries.

### Databases and search strategy

We searched PubMed, EMBASE, LILACS, Scopus, Econlit, PsycINFO, Global Health and Global Index Medicus for original papers, working papers, reports, theses, and official documents. We included search terms in Portuguese, Spanish and English as they comprise both countries’ official and second languages (See the Search Strategy in S2 Table). A simplified search was also performed on Google Scholar. We hand-searched for key documents in the Brazilian and Ecuadorian Ministries of Health and Economic Affairs, the United Nations (UN) Economic Commission for Latin America and the Caribbean (ECLAC) and Pan American Health Organization (PAHO), Inter-American Development Bank, World Bank and Organisation for Economic Co-operation and Development (OECD) Latin America & the Caribbean. Finally, we checked the reference list of all identified studies and reports for additional references.

### Selection of studies, data extraction and synthesis

Studies identified were compiled, deduplicated and screened with Rayyan by two researchers in duplicate [17]. In cases of disagreement during title/abstract or full-text screening, the two researchers discussed the conflict. If consensus could not be reached, a third senior researcher was consulted. The key guiding principle for conflict resolution was strict adherence to the inclusion and exclusion criteria defined a priori in the OSF protocol. These criteria included specific definitions regarding target populations (children and adolescents, elders, people with disabilities, and their caregivers), study design (quantitative or qualitative evaluations), comparison groups (evaluation studies without a counterfactual group), and the focus on one of the seven selected social protection programs. Additionally, two experienced researchers from Brazil and one from Ecuador searched the grey literature of their country.

The core data of each selected social protection program was extracted by one policy specialist for each country (i.e., investigators of this scoping review) [18] following an adapted version of the TIDieR-PHP template [19]. We extracted (i) basic information on each program, including the name of the program, main aim, intended short, medium and longer-term impacts, dates that the program was implemented and ended; (ii) information on program delivery, including mechanism of assessing eligibility, eligibility, enrolment, benefits, conditionalities, infrastructure and support provided as part of the intervention, combination with other programs, delivery, ways of monitoring eligibility and conditionalities as well its sanctions when failing to meet conditionalities; and (iii) Implementation characteristics, including who is responsible for the program implementation in each country, speed and coverage of implementation, and planned and unplanned variations.

Data was extracted by one researcher using a standardised form containing data on the aims of the study, study design, study setting, year, population and size, methodology, intervention and comparator descriptions, outcome description, effect direction and main results.

We followed the Synthesis Without Meta-analysis (SWiM) guidelines to report our findings [20] using tables, a narrative synthesis of the available evidence by program and the planned (or intentional) and unplanned (not intentional) outcomes. Planned and unplanned outcomes were defined based on available data on the program’s aims (official documents) and targeted populations. The amount of evidence was summarised into few, some or large, depending on the number of studies and their design, and evidence was further grouped into those with longitudinal or experimental/quasi-experimental designs.

Importantly, throughout the manuscript, we refer to ‘programs’ as the operational expressions of broader ‘policies’. For instance, BPC is not a program but a policy included in Brazilian legislation. However, we referred to BPC as a program. Additionally, we use ‘effects’ to generally describe observed specific associations between programs and outcomes.

The visualisation was done in R Studio, version 4.2.2, using “ggplot”. We used discussions with health and social protection managers to translate the available evidence, and to inform future research.

## Results

### Search results

**Fig 1:**
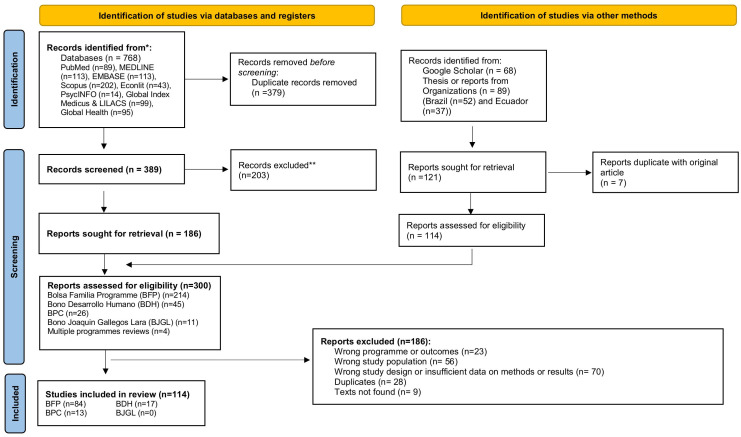
PRISMA flowchart for the selection of the studies.

We summarized data of all the programs and reviewed 114 studies, of which 84 were on the Bolsa Familia Program from Brazil, 17 on Bono de Desarrollo Humano from Ecuador and 13 on the Continuous Cash Benefit from Brazil (See Fig 1 and Table 1 for programs descriptions). No studies were identified for Bono 1000 días, Pensión Mis Mejores Años (previously named Pensión para adultos mayores), Pensión Toda una Vida (previously named Pensión para personas con discapacidad) or Bono Joaquín Gallegos Lara.

**Table 1:** Social protection programs eligibility criteria, benefits, conditionalities and main variations over time.

Social protection programs benefiting:	Aim, start date and current status	Eligibility	Benefits	Conditionalities	Main variations
***Children from families experiencing poverty***					
**Bolsa Familia Programme (BRAZIL)**	Aim: To fight intergeneration poverty by guaranteeing a basic income for families in poverty, while facilitating families’ access to basic rights such as health, education and social assistance. Start date: Created in 2003 but implemented in January 2004. Status: ongoing	From 2004-2020: Families living in extreme poverty (monthly per capita household income ≤BRL89 in 2020 (approximately USD18)) or poverty (monthly per capita household income ≤BRL178 in 2020 (approximately USD36)) and that have a pregnant, breastfeeding woman or child/ adolescent in the household. Individuals must be registered in the Brazil’s National Registry for Social Programs ‘Cadastro Único’ (CadUnico). 2022-now: Families with monthly per capita income up to BRL218 (USD44)	In 2020, extremely poor families received a fixed benefit of BRL89 plus supplementary amounts for pregnant women, children and adolescents. Families defined as poor receive the supplements for pregnant women, children and any adolescents in the household, but not the fixed benefit. Additional benefits are given to families to overcome extreme poverty. As in 2024, the benefit is BRL600 plus BRL50 per child or adolescent up to 18 years old or pregnant person in the household.	Health conditionalities include completion of regular Brazilian vaccination schedule, regular health check-ups for growth monitoring for children ages 0–7 years old, and pre- and post-natal checkups for pregnant women. Education conditionalities for children or adolescents include minimum of 80% of school attendance.	Several changes were mainly implemented in the value of the Benefit to adjust for inflation and in the number of supplementary benefits per person. In 2011, the maximum number of supplementary benefits allocated per family went from five to seven. In 2012, it was also implemented an additional amount given to families to overcome extreme poverty (Benefício para Superação da Extrema Pobreza na Primeira Infância – Brasil Carinhoso). In 2020 families receiving Bolsa Familia started automatically receiving a mínimum income for COVID-19. During 2021, Bolsa Familia was provisionally replaced by “Auxilio Brasil”[135,136]. In 2022 the programme was simplified to include all families living into poverty without further requirements [137,138].
Bono de Desarrollo Humano (ECUADOR)	Aim: To address vulnerabilities related to the economic situation of families in poverty or extreme poverty conditions. Start date: 2003 Status: ongoing	Family units with children up to 18 years of age in situations of poverty or extreme poverty according to the current Social Registry	USD. 55/ month Variable component, consisting of an additional USD 30.00 for each child under 5 years old, with a maximum of 3 children; and an additional USD 10.00 for each child aged 5 years or older but under 18 years, with a maximum of 3 children.“	Pregnant women must undergo five prenatal medical check-ups. Children between 1 and 4 years old must have at least 4 medical check-ups per year. Children between 6 and 17 years old must have at least one medical check-up per year. Children must be enrolled and regularly attend classes at the basic general education and high school levels, as appropriate. Children under 15 years of age are prohibited from engaging in any type of work.	2013: There was a institutional restructuring to coordinate social protection and social mobility policies; in this context, the Deputy Ministry of Non-Contributory Assurance and Social Mobility (VAMS) was established within the MIES, which oversees the execution of the BDH and other related programs. 2014: In the face of reduced fiscal capacity due to the drop in oil prices, stricter eligibility conditions were introduced to redirect social programs towards the family in extreme poverty, and the largest exclusion strategy from the program was implemented. 2018: A variable amount up to USD150 has been given to families in extreme poverty according to the number of children under 18 years of age 2022: The benefit of 50 dollars since 2013 was increased to 55 dollars as compensation for the global economic crises and COVID-19 pandemic.
Bono 1000 días (ECUADOR)	Aim: To ensure a minimum floor of consumption, as well as promoting the use of health and child development services, and raising awareness about maternal and child nutrition and good health practices. Start date: 2022 Status: ongoing	Pregnant women and children up to two years of age experiencing poverty and extreme poverty according to Social Registry	USD. 60/month $50.00 will be provided as the unconditional monthly component, while the remaining $10.00 will be part of the conditional component.	Pregnant women should attend at least one check-up every three months throughout the 9 months of pregnancy. Children should undergo at least 6 growth check-ups during the first year of life and at least 4 check-ups in the second year of life. Birth registration must be completed before the age of 45 days.“	No variations
***Elders and people with disabilities experiencing poverty***					
Beneficio de Prestacao Continuada (BPC) (BRAZIL)	Aim: The benefit is included in the constitution in Article 203 as to “guarantee of a minimum wage monthly benefit to the disabled person and the elderly who prove that they do not have the means to provide for their own maintenance or have it provided by their family”. Start date: Created in 1996 but implemented in January 2006. Status: ongoing	Elderly people and people with disabilities in a situation of social vulnerability. 1) Income eligibility include per capita monthly income equal to or less than ¼ of the minimum wage (considering the family group) (i.e., BRL 1320 or USD 264 in 2023). AND 2) Elderly, aged 65 (sixty-five) years or more; or person with a disability, of any age. People with disabilities are those who may encounter limitations in social participation compared with those without disabilities, due to the interaction between their physical, intellectual and sensory impairments and environmental barriers (Decree 13.146/2015). More specifically defined as: (i) someone who has long-term impairments of a physical, intellectual or sensory nature, which, in interaction with various barriers, may obstruct their full and effective participation in society with other people; or (ii) who has health conditions that functional limitations for a minimum period of 2 (two) years (Art 20, decree 8742/93 and 12435/2011).	One minimum wage - BRL 1,212 (2022). Since 2021, BPC also includes the “auxilio-inclusao” which also pays half the minimum wage to people with disabilities who are or have been, in the last 5 years, beneficiaries of the BPC and have started a formal job with remuneration of up to 2 minimum wages. The benefit cannot be accumulated with another Social Security benefit.	None	2003: The age for accessing BPC reduced from 67 years to 65 years (Law n. 10,741). 2007: introduction of the need to proof of income below ¼ of the minimum wage per capita family, and the disabled person should be submitted to analysis of the medical expertise of the INSS. 2009: Start of the use of WHO International Classification of Functioning, Disability and Health – ICF (2001) instead of the former definition of “presence of long term disability that generates impossibility to work using the ICD-10” as ways to assess and define eligibility 2018: It became mandatory to be registered in CadUnico to receive the benefit. Before that date, this was not a requirement. 2021: Includes income threshold as up to ½ minimum wage in case of higher degree of disability, dependence on third parties and dependence on social and health expenses not covered by the Brazilian Universal Social and Health care (Decree 14.176/2021). Also implement that: (i) not inclusion of expenses with health treatments, special foods and diapers for the elderly or disabled person from the income calculation in case they are not provided by the public health system; (ii) application of an “average standard” to the social evaluation - that is, in cases where the social evaluation was classified as “standard” the benefit cannot be rejected; (iii) precautionary block, which prevents the transfer of the benefit amount when there is suspicion of fraud or irregularity in granting the BPC, which the INSS has to analyse in up to 30 days.
Pensión para adultos mayores^**1**^ (ECUADOR)	Aim: To address economic shortcoming and expenses associated with vulnerabilities that are acccentuated by age. Start date: 2003 Status: 2023	Elderly people (≥65 years) who do not have access to contributory social security coverage and previously eligible based on the 2014 Social Registry information, until their information is updated.	USD 50/month	None	The year 2019 marked the beginning of the gradual phase-out of the “Pensión para adultos mayores”, with its recipients being progressively transferred to the “Pensión Mis Mejores Años”. Since 2013, the benefit has increased from 35 to 50 dollars.
Pensión Mis Mejores Años^**1**^ (ECUADOR)	Aim: To address economic shortcoming and expenses associated with vulnerabilities that are acccentuated by age. Start date: 2017 Status: ongoing	Elderly people (≥65 years) in situations of poverty and extreme poverty according to the current Social Registry (2018)	USD. 100/month	None	No variations
Pensión para personas con discapacidad^**1**^ (ECUADOR)	Aim: To address economic deficiencies and expenses incurred by individuals with a disability condition. Start date: 2003 Status: 2023	People with disabilities greater than 40%,not covered by the contributory system and previously eligible based on the 2014 Social Registry information, until their information is updated.	USD. 50/month	None	The year 2019 marked the beginning of the gradual phase-out of the “Pensión para Personas con Discapacidad”, with its recipients being progressively transferred to the “Pensión Toda Una Vida” Since 2013, the benefit has increased from 35 to 50 dollars.
Pensión Toda una vida ^**1**^ (ECUADOR)	To address economic deficiencies and expenses incurred by individuals with a disability condition. Start date: 2019 Status: ongoing	People with disabilities greater than 40% in poverty and extreme poverty according to the current Social Registry (2018).	USD. 100/month	None	No variations
Bono Joaquín Gallegos Lara (ECUADOR)	To address economic deficiencies and expenses incurred by individuals with a disability condition. Start date: 2010 Status: ongoing	People with physical, intellectual, and psychosocial disabilities (≥65% score of intellectual and psychosocial disabilities or ≥75% score of physical disability), with catastrophic, rare and orphan diseases and children under 14 years of age living with HIV-AIDS, in critical socioeconomic condition	USD. 240/month	None	No variations

^**1**^ Pensión Mis Mejores Años and Pensión para adultos mayores coexisted until November 2023. The same applies to the programs “Pensión para personas con discapaciad y Pensión Toda Una Vida”. In December 2023, Pensión para Adultos Mayores and Pensión para personas con discapacidad programs ceased to exist because individuals who were still living in poverty and extreme poverty, according to the latest evaluation, were transitioned to the Pensión Mis Mejores Años program and Pensión Toda una Vida, respectively. In summary, since 2009, the “Pensión para Personas con Discapacidad” was the only one in place, targeting individuals living in poverty and extreme poverty according to the 2014 Social Registry and with a 40% disability. In 2019, the “Pensión Toda Una Vida” pension was established for individuals with a 40% disability but classified as poor and non-poor according to the updated 2018 Social Registry. Consequently, the year 2019 marked the beginning of the gradual phase-out of the “Pensión para Personas con Discapacidad”, with its recipients being progressively transferred to the “Pensión Toda Una Vida”. In November 2023, the “Pensión para personas con discapacidad” was definitively closed, leaving only the “Pensión Toda Una Vida” pension in place. On the other hand, The “Bono Joaquin Gallegos Lara” is intended for people with severe disabilities of 70% or more, and it also covers individuals with catastrophic, orphan, or rare diseases.

### Data availability, approaches and study designs

#### Bolsa Familia Program.

The majority of studies used survey data collected once or repetitively across several study years (See Table 2 and S3 Table and S4 Table), whilst some studies used individual-level primary or secondary data on health linked to socioeconomic data from the Unified Registry for Social Programs (Cadastro Unico para Programas Sociais, CadUnico) that is the main mechanism of accessing eligibility for social programs in Brazil. Study designs included (i) an ecological approach with municipality-level data over time using data on BFP coverage and health or SDH data in Brazilian municipalities since BFP implementation; (ii) cross-sectional studies using individual-level data collected through small studies or survey data to analyse the association between BFP and health or SDH at one-time point (i.e., without taking into account when BFP benefit started); (iii) difference-in-difference designs using individual-level data collected through survey data to analyse the association between BFP and health or SDH but taking into consideration multiple time points; (iv) regression discontinuity designs using income as a cutoff point, or (v) cohorts with individual-level data with detailed information on benefit receipt (e.g., cohorts containing BFP data or cohorts of linked data).

**Table 2:** Main sources of data, outcomes measured, indication of overall results and amount of evidence.

Country/programme and characteristics	Available data to study*	Planned and unplanned effects	Outcomes	Overall results	Amount of evidence 1 – few evidence 2 – some evidence 3 – large evidence
***Brazil***					
Bolsa Familia Programme (BRAZIL)	Evaluation of the Impact of Bolsa Familia Program survey (Avaliação de Impacto do Programa Bolsa Família, AIBF) Brazil’s National Household Sample Survey (Pesquisa Nacional por Amostra de Domicílios, PNAD) Unified Registry for Social Programs (Cadastro Unico para Programas Sociais) linked with other data sources Cobertura munic: Ministério de Desenvolv. Social e de Combate à Fome (MDS). Family Budget Survey (Pesquisa de Orcamentos Familiares, POF) Pesquisa Nacional de Saúde (PNS)	Planned effects	Mortality and morbidity of children: all-cause neonatal, infant and child mortality, diarrhoea episodes, diarrhoea hospitalisations and mortality	Benefit	○○ [62,91–98]
			Perinatal health (children): preterm or low birth weight	Benefit	○ [139,140]
			Perinatal health (mothers): maternal mortality	Benefit	○ [89–91]
			Healthcare utilisation: frequency of healthcare utilisation and monitoring and vaccination	Varied evidence	○○ [70,81,85,96,99–101]
			Nutrition and growth: food security (food expenditures, frequency and consumption, food insecurity index), nutritional status (linear growth, weight, height for age, weight for age, weight for height, Body Mass Index), anaemia, vitamin A deficiency, hospitalisations by anaemia, overweight, obesity,	Varied evidence	○○○ [22,57–88]
			Education: school enrolment and attendance, dropout rates, grade progression, grade repetition or age-grade distortion	Varied evidence	○○ [35–46,70]
			Child labour: number of hours worked, informal and informal work	Varied evidence	○○ [41,43,47–51]
			Poverty, work and social mobility: income and work or type of work (mothers or caregivers), economic and social inclusion, poverty, social cohesion, social vulnerability, family expenditures in school materials and health	Benefit	○○ [52–56]
		Unplanned effects	Oral health: prevalence and severity of dental caries	Varied evidence	○ [58,102–105]
			Mental health: mental health in children and adolescents	Varied evidence	○ [106]
			Infectious diseases of poverty: leprosy incidence and cure	Benefit	○ [107,108]
			Healthcare utilization and morbidity (caregivers): cervical cancer screening, NCD among women in reproductive age, adolescent pregnancy	Benefit	○ [109–111]
**Beneficio de Prestacao Continuada (BPC)**	Sistema Único de Informações de Benefícios (SUIBE) Programa BPC na Escola (Programa de Acompanhamento e Monitoramento do Acesso e Permanência na Escola das Pessoas com Deficiência Beneficiárias do Benefício de Prestação Continuada da Assistência Social) Pesquisa Nacional por Amostra de Domicílios, or PNAD	Planned effects	Labour: number of hours worked (older adults)	Benefit	○ [127]
			Health: nutrition, food security, mortality and healthy-life (older adults)	Benefit	○○ [129–131]
		Unplanned effects	Income: health inequality	Benefit	○ [128]
			Education: school attendance of family members of older adults	Benefit	○ [127]
			Child labour: labour of younger family members of older adults	Benefit	○ [127]
			Mortality and morbidity (all age groups in the general population)	Benefit	○ [131]
***Ecuador***					
**Bono de Desarrollo Humano (BDH)**	Living Conditions Survey (Encuesta de Condiciones de Vida) National Employment, Unemployment, and Underemployment Survey (Encuesta Nacional de Empleo, Desempleo y Subempleo) Ser Bchiller y Encuesta de Factores Asociados Encuesta de Situación Socio-económica de los Hogares Registro Interconectado de programas sociales Base de datos de defunciones y egresos hospitalarios (INEC)	Planned effects	Nutrition and growth: breastfeeding, food insecurity, height-for-age z score, haemoglobin levels.	No evidence	○○ [112–114]
			Morbidity and mortality of children: all cause under-five mortality	Benefit	○ [115]
			Education: school attendance, enrolment and educational delay	Varied evidence: effect concentrated in extremely poor people	○○○ [116–119]
			Poverty and social mobility: gini index, social mobility, poverty level	Benefit	○○○ [122–125]
			Child labour	Benefit	○ [118]
		Unplanned effects	Cognitive skills: language and math skills	Varied evidence: effect on very young children (12–35 months old) in rural areas but no effect on school children and adolescents	○○ [114,120,122]
			Healthcare utilization and morbidity (caregivers): gender equality measured as the well-being of mothers	Harmful effect	○ [126]
**Bono 1000 días**	NA	NA	NA	NA	NA
**Pensión para adultos mayores**	NA	NA	NA	NA	NA
**Pensión Mis Mejores Años**	NA	NA	NA	NA	NA
**Pensión para personas con discapacidad**	NA	NA	NA	NA	NA
**Pensión Toda una vida**	NA	NA	NA	NA	NA
**Bono Joaquín Gallegos Lara**	NA	NA	NA	NA	NA

*Without considering primary data.

#### Bono de Desarollo Humano.

Most studies on the BDH analysed secondary data from surveys, and few studies used data from the Social Registry or the information system of the Ministry of Social Development (see S5 Table). Individual studies mostly used regression discontinuity and the Social Registry Index score to assign treatment and control groups. Additionally, two studies were randomised clinical trials, which involved selecting parishes whose households had not received the transfer at baseline but were eligible to receive it to constitute the intervention and control groups. Finally, only one study used a mixed ecological design with counties as the unit of analysis for the period from 2009 to 2014.

#### Continuous cash benefit.

Similar to BFP, BPC studies on implementation mainly mixed qualitative and quantitative analysis of survey and primary collected data (see S6 Table). Only one study used de-identified data from Single Registry for Beneficiary Information (Sistema Único de Informações de Benefícios – SUIBE), which is the primary registry used by the Instituto Nacional do Seguro Social (INSS, Brazil’s National Institute of Social Security) to assess potential beneficiaries’ eligibility for BPC along with Cadastro Unico. To analyse the effects, there was one cross-sectional survey data from PNAD or semi-structured interviews with beneficiaries and with beneficiaries and stakeholders (i.e., primary data), one study using difference-in-difference designs using municipal-level data BPC coverage data over time, and one study applying regression discontinuity design with age as the cut-off point to survey data containing family level BPC benefit receipt. Similar to BDH, no study evaluated the effects of BPC using linked individual records.

### Programme implementation

#### Bolsa Familia Program.

We found three studies highlighting aspects such as programme implementation, coverage and focus of the programme in those groups, of which one also explored BFP effects on health. In 2007, three years after BFP implementation, BFP coverage (% of eligible people receiving the benefit) already reached 77.6% of eligible families in Brazil, which at that time included families with monthly per capita income < 100 Brazilian reais (BRL) [21]. By analysing BFP coverage and focalisation (% of people receiving the benefit that are eligible) among families enrolled in the “Pelotas Birth Cohort” at birth (i.e., in 2004) and at age 6 years (in 2011) [22], it was found that considering the same income threshold (i.e., BRL100 in 2004 and BRL140 in 2011), BFP coverage increased from 42.8% in 2004 and 70.9% in 2010, while the focalisation of the programme decreased from 77.9% in 2004 to 32.4% in 2010 [22]. However, qualitative research suggested a lack of trained personnel to effectively manage the program and ensure compliance with its conditionalities [23]. Those include shortages of trained personnel to operate the program, lack of intergovernmental cooperation and intersectoral coordination, insufficient space for experience sharing, disorganisation and poor and coercive management of conditionalities (e.g., leading to temporary disruptions unrelated to family circumstances but rather to the monitoring system itself), and poor attendance and effectiveness of the PBF Social Control Commission [23].

#### Bono de Desarollo Humano.

We found only two studies focusing on implementing the BDH in Ecuador. A first study conducted by the ECLAC suggested a decrease in the coverage of social programs, particularly in Ecuador, since the early 2000s[24]. In Ecuador, the coverage of CCTs dropped from 44% in 2000 to 13% in 2015, a decline attributed to changes in the Human Development Bonus (BDH) program starting from 2013 [24]. Due to fiscal constraints, the target population shifted from people living in poverty and extreme poverty to only those in extreme poverty, and a process was introduced to phase households out of the program. The BDH budget also decreased from 1.062 million dollars in 2013–651 million in 2015 [24]. A study using a mixed-method approach, incorporating both qualitative and quantitative analysis, assessed the effect of costs on the take-up of BDH and suggested that travel costs, personal identity stigma, and dissatisfaction with the government pose important obstacles to take up, after controlling for program design and household poverty [25]. Programs’ take-up was lower in households considered to be “not poor” than among households considered “more or less poor”, “poor”, or “very poor” (43% vs 65% in other households) [25]. Finally, interviews with BDH-eligible Ecuadorians, particularly in remote areas, suggested that the lack of documentation (i.e., birth certificates, identification documents, among others) could be a major obstacle to take up [25].

#### Continuous cash benefit.

We found eight studies looking at the implementation of the BPC in Brazil, including (i) studies of take-up and coverage [26], (ii) on the claiming process and administration [27–29], (iii) on the effect of introducing after the introduction of the International Classification of Functioning, Disability and Health (ICF) [30,31], and (iv) studies of participation in schooling by BPC beneficiaries [32,33].

Between 2004–2014, the number of people applying for BPC regardless of their income was 2% of the total population aged ≥65 and 0.25% among people under 65 with disabilities (i.e., people reporting great difficulty or impairment to vision, hearing, or mobility, as well as mental disability) [26]. The proportion of BPC benefits granted through legal concessions (i.e., denied by INSS but further approved after judicialisation) was 17% of all benefits for people with disabilities and 4% for elders [26]. Studies pointed to the lack of formal institutionalised collaborations between INSS and municipal social and health management, coordination or monetary incentives to adhere to federal policies to reduce barriers to people accessing BPC [28], with low participation of medical experts (3.3% of benefits between 2006 and 2015) and reduced participation of social welfare professionals in the process of granting the BPC during the same time [29]. In addition, the program’s complexity, the lack of information by applicants and health professionals, the nature of the target population and the lack of interaction between social and health services pose several barriers to its access [27,28].

After the introduction of the ICF, which served as the basis for the preparation of the Brazilian assessment instrument used by the INSS since 2009: the Brazilian Functionality Index (IF-Br) [34], studies found a 22% increase in the percentage of BPC concession for eligible people with disabilities [30], but with differences depending on the age group [30]. In addition, from 1998 to 2014, the incorporating the new classification, the speed of granting benefits has not increased and BPC may not have been able to reach all the target population, with men 1.5 times more likely than women to have their application deferred [31].

Finally, a study looking at participation and barriers in schooling by BPC beneficiaries showed that, in 2008, 7.9% of children aged 12 never attended school, of which 71,6% were because the caregivers never looked for access for children at school [32]. In 2012, a second study found that the number of individuals under 18 years who are beneficiaries from BPC who were not enrolled at a school was 28.84% in 2012 [33]. Both studies pointed out that children with disabilities face difficulties attending school [32], including those related to care of the individual, insertion of children and adolescents into teaching and learning activities and lack of public policies necessary to make it possible to stay at school [33]. In addition, among caregivers who tried to enrol their children at school, 18.1% cited that schools refused to enrol the children/adolescent [32].

### Effects on socioeconomic determinants of health and health

#### Bolsa Familia Program.

**Fig 2:**
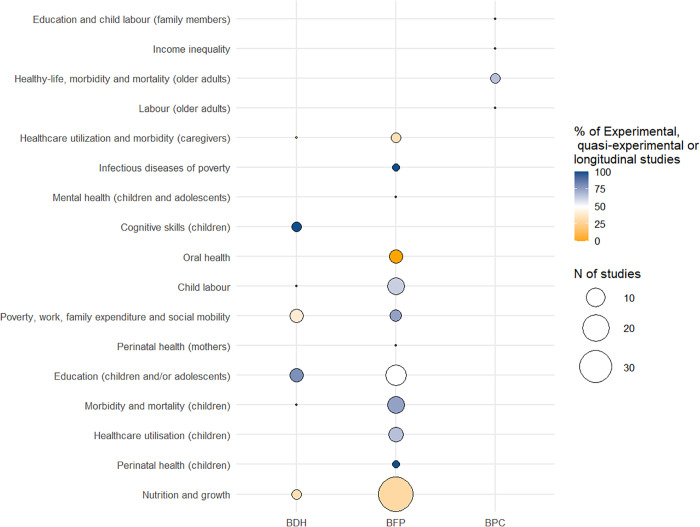
Evidence map for the effects of social protection programs targeted to children (Bolsa Familia Programme – BFP, or Bono de Desarrollo Humano – BDH) and to elders and people with disabilities experiencing poverty (Beneficio de Prestacao Continuada – BPC) on health and social determinants of health of children.

We included 81 studies looking at the effects of BFP on health and socioeconomic determinants of health, of which 22 investigated the effects of BFP on the socioeconomic determinants of health and 59 the effects of BFP on health outcomes (See Table 2, 23 Table, S4 Table and Fig 2). We considered as planned effects those that were aligned to the program objectives, such as the effects of the cash on improving food security and nutrition, reducing poverty, fomenting education of children and reducing child labour, fomenting intergenerational social mobility and wealth; and those directly affected by conditionalities such as health indicators of children under 5, perinatal outcomes of mothers and newborns, and education of children and adolescents. We considered the effects on other health outcomes as unplanned (See Table 2).

Twelve studies analysed the effect of BFP on educational indicators such as school enrolment and attendance, dropout rates, grade progression, grade repetition or age-grade distortion [35–46], of which one was a systematic review [45]; and one paper investigating if BFP could mitigate shocks that might affect educational outcomes, such as rainfall [41]. Seven studies analysed the effect of the program on child and adolescent labour [41,43,47–51]. Five studies looked at the effect of BFP on the work or employment of caregivers, poverty, social cohesion or other markers of improved socioeconomic conditions and social mobility [52–56], of which one investigated social inclusion, exclusion and social cohesion using a qualitative approach [53]. Although most studies attempted to apply quasi-experimental designs (i.e., including regression discontinuity around the income threshold or propensity score methods), only two studies extracted information before and after receiving BFP [43,52].

Effects of BFP on health are analysed in 59 studies, but few have drawn on longitudinal data or applied quasi-experimental designs to find comparable interventions and comparison groups (See Fig 2). Regarding the planned effects, nutritional and growing monitoring outcomes were the most frequently studied [22,57–88]. Still, the evidence of its beneficial effects on reducing malnutrition and improving growth outcomes was not consistent across studies, age groups and study designs. This was further evidenced in a 2009 systematic review, which included data from social protection programs in Brazil, Ecuador, and other countries [87]. Other planned effects of BFP included maternal mortality [89–91]; mortality and morbidity of children, including for different age groups and by causes such as diarrhoea [62,91–98], healthcare utilisation, monitoring and vaccination [70,81,85,96,99–101]. In terms of unplanned effects, some studies looked at oral health of beneficiaries and non-beneficiaries of BFP [58,102–105], one looked at mental health of children of beneficiary families of BFP over time [106], two studies looked at the effect of BFP on leprosy incidence and treatment outcomes among children/adolescents under 15 years [107,108], and three looked healthcare of caregivers or women at reproductive age, including cervical cancer screening, non-communicable diseases prevalence and adolescent pregnancy (i.e., aged 15–18 years) [109–111]

#### Bono de Desarrollo Humano.

Fifteen studies investigated the effects of BDH on SDH or health outcomes, four assessing their effects on health and eleven on SDH (See S5 Table and Fig 2). Twelve were observational studies data on coverage of BDH and health or educational indicators, using either households or areas as the unit of analysis; two were randomized control trials using primary data and one was a qualitative study. Among the studies that assessed the effects on health or SDH (N = 15), the majority (93%) were quantitative studies and assessed the effect on nutrition and growth (e.g., exclusive breastfeeding, food insecurity, height-for-age z-score and haemoglobin concentration) [112–114], one on morbidity and mortality of children by all-causes and specific causes [115], four on school enrolment and delay [116–119], three on cognitive achievement and skills [114,120,121], four on poverty and social mobility [122–125], and one on child labour [118]. The only qualitative study evaluated the effect on women’s rights, in terms of their quality of life, access to basic social services, visibility, and empowerment. Similar to the Brazilian BFP, BDH includes health and education conditionalities for children and breastfeeding women (See Table 1 and Table 2). Therefore, observed effects on child nutrition, development, morbidity and mortality and education of children were considered planned effects; whereas more general effects on poverty reduction and gender inequalities were considered unplanned effects (See Table 2).

Most studies effects evaluated the effect of BDH on health or SDH indicators used regression discontinuity designs based on the unified vulnerability assessment index (i.e., SELBEN points cutoff point for eligibility of 50.65 [113,117,120]), or the Social Registry scores (cutoff points for eligibility of 34.68). Some were also randomized trials with baseline and follow-up surveys conducted by the World Bank and the government of Ecuador [114,116,118], used a difference-in-difference approach with individual-level data [117,122,124], and one an ecological study applying a time series in Ecuadorian counties between 2009 and 2014 [115]. Two descriptive studies used ENEMDU (Encuesta Nacional de Empleo, Desempleo y Subempleo), and looked at the effects of receiving BDH on poverty [123]; and estimated what the poverty rates would have been in the absence of the program [125]. Finally, one study evaluated the effect of the BDH on gender inequalities using a qualitative methodology, including focus groups, workshops with stakeholders, and semi-structured interviews in three counties of Ecuador [126].

#### Continuous cash benefit.

Five studies analysed the effects of BPC on health or SDH (See S6 Table and Fig 2). Two studies evaluated the effects of BPC on the socioeconomic determinants of health (i.e., municipality health inequality measured by the Gini Index and individual and labour force participation in the month or week before the benefit receipt of the beneficiary and co-residents aged 18-49y, 18-29y and of children aged 10-15y (i.e., child labour) and school attendance of children aged 10-15y) [127,128] and three evaluated the effects on healthy (one on healthy life expectancy [129], one on nutrition and food security [130] and one on all cause hospitalization and mortality) [131] (See Table 2). The latter also evaluated the effect of BFP but included both programme coverages in the same analysis [131]. The definition of beneficiaries in each study varied but only one contained information on benefit receipt, while the others were either conducted at ecological level or based on proxies such as age and income. Only one study applied a difference-in-difference approach using repetitive individual level measurements from PNAD data for 1995 and 2006 [128], while two explored the age as a cutoff using a regression discontinuity design (RDD) [127,130].

## Discussion

In this study, we have summarised the scope of evidence on the implementation and effects on the socioeconomic determinants of health and health conditions of key social protection programs or policies targeting children, pregnant women, elders and people with disabilities experiencing poverty in Brazil and Ecuador. In Brazil, we found substantial literature on the planned effects of the Brazilian conditional cash transfers targeting children and pregnant women but little evidence for unplanned effects on those groups. In addition, we found very little evidence of the effects of the continuous cash benefit targeting elders and no evidence for people with disabilities. In Ecuador, only the BDH was evaluated. There were few individual-level studies, mostly in the early years of the programme implementation, with only ecological data available to study long-term effects.

When looking at social protection for children and pregnant women, the Brazilian BFP programme has been extensively researched regarding its effects on reducing poverty and inequality, improving the socioeconomic determinants of malnutrition, and morbidity and mortality among children and mothers. However, only few studies adequately compare beneficiaries with non-beneficiaries who were eligible for the programme, suggesting that further research should use longitudinal studies and with adequate intervention and comparison groups to provide better evidence regarding possible causal links between the intervention and the studied outcome. The BDH has been evaluated using individual data in the first years of implementation in villages that were randomised to receive the intervention. Long-term or unplanned effects of BDH have only been evaluated at the ecological level, which limits the scope of analysis for specific subgroups. Our review also highlights that while there is some evidence of the BDH program having positive effects, particularly on reducing under-5 mortality and poverty, its effects on other health and educational outcomes are less conclusive. There is no individual-level data available to understand further the overall unplanned effects and long-term planned effects of BDH on the SDH and health outcomes (See Box 1).

In relation to implementation, we found that successful delivery of both BFP and BDH depends on the presence of complementary, but relevant initiatives [132], such as better coordination between the bodies responsible for providing the cash with those providing health and education conditionalities. For instance, whilst BDH enables the maintenance of a certain level of consumption for the poorest households [126], the pressure of the conditionalities, including the penalty of withdrawing the cash transfer, can be a source of daily confrontations within households that undermine women’s abilities to achieve their own well-being and of their children [126]. These tensions in family relationships can have negative effects on the emotional well-being of mothers and children that can, in many cases, nullifies the potential positive impacts of the transfer [126].

Therefore, the absence of coordinated actions or programs that work in conjunction with cash transfers limits the effects of programs such as the BDH on improving the quality of life and access to social services [126]. Social protection programs are often implemented alongside other social and health interventions, but there is limited research on how these programs interact and their combined effects on health outcomes. Future studies should investigate the interaction between different programs to identify potential synergies and optimize their impact.

We found no studies looking at the effects of social protection for elders and people with disabilities in Ecuador. However, the Brazilian BPC was found to have the potential to alleviate poverty and improve the quality of life of elders and people with disabilities experiencing poverty in Brazil, given its targeted eligibility and high levels of benefits. The few studies available on the program indicate that BPC can reduce income inequality, improve the quality of life and increase participation in work and education among eligible elders and their household members. However, limited research is available on the effects of BPC on morbidity and mortality in elders or people with disabilities, suggesting that further studies of both its broader planned and unplanned effects would be useful (See Box 1). Finally, although BPC coverage and instruments that adequately measure disabilities have improved over time, the barriers to accessing BPC can limit the planned and unplanned effects of BPC on the social determinants of health and the health of targeted populations.

Our scoping review has some limitations. First, we might not have captured all the literature about program implementation or effects, especially in the context of Brazil, as we may not have identified studies that were not focused on children, older people, people with disabilities and their caregivers. Second, our search might not have captured the effects of global economic crises and the COVID-19 pandemic on the implementation of social programs and their impact on population well-being. However, we conducted a comprehensive search, including grey literature, and summarised a large amount of evidence regarding the seven social protection programs focusing on the most vulnerable people in Ecuador and Brazil.

## Conclusions

In conclusion, our scoping review identifies the need for more research on the unplanned effects of BFP on health outcomes, as well as on the implementation and planned and unplanned effects of BPC, BDH, and other social protection programs focusing on elders and people with disabilities in Ecuador and Brazil. Most importantly, the lack of individual-level data and studies investigating planned and unplanned health effects in Ecuador prevents us from understanding its long-term effects. Because randomized controlled trials are often not feasible, studies could more frequently and better utilize quasi-experimental designs, simulations, and forecasting models to determine the most impactful and cost-effective interventions. In addition, we highlight the importance of more standardised approaches to investigating the effects of social protection on health outcomes, with more detailed socioeconomic data that allow the construction of better and more comparable control groups. Recent legal and policy frameworks in both Brazil (Presidential Decree No. 11.353/2023 [133] and a proposed bill PL 3083/2022) and in Ecuador’s constitution [134] have reinforced the importance of monitoring and evaluating social programs aiming to promote transparency, efficiency, and alignment with stated objectives. However, our review shows that systematic evaluations remain scarce, often constrained by limited data availability, methodological capacity, and political or institutional priorities that emphasises program expansion or operational continuity over rigorous impact assessment. Furthermore, qualitative research should play a complementary role in supporting and interpreting quantitative findings.

**Box 1. Suggested priorities and potentialities to study the effects of social protection programs on health or Socioeconomic determinants of health outcomes based on this review and discussions with stakeholders.**

**Brazilian Bolsa Familia Programme (BFP)**

- Unplanned effects on infectious diseases of poverty in targeted groups (e.g., infant tuberculosis)
- Unplanned effects on violence and pregnancy in children and adolescents
- Effects of changes in the BFP conditionalities monitoring on food and nutritional insecurity, growth curve and others
- Mitigation effects of climate change on health events
- Interaction of BFP with other social protection and health programmes

**Brazilian Beneficio de Prestacao Continuada (BPC)**

- Effects on all-cause and cause-specific mortality and morbidity of older adults
- Effects on all-cause and cause-specific mortality of people with different grades and types of disabilities
- Unplanned effects on health status (morbidity and mortality) of people living with BFP beneficiaries
- Interaction of BPC with other social protection and health programmes

**Ecuadorian Bono de Desarrollo Humano (BDH)**

- Unplanned effect on the mental health of children and their caregivers, health behaviours and gender dynamics.
- Effects on premature and all-age cardiovascular mortality
- Effects on maternal mortality
- Effects on mitigating the impact of economic crises/ economic downturn.
- Interaction of BDH with other social protection and health programmes

## Supporting information

S1 Table Eligibility criteria for selecting papers. (DOCX)

S2 Table PIIE (Population, Intervention, Implementation and Effects) search terms. (DOCX)

S3 Table Studies on the effects of the Bolsa Familia Programme on socioeconomic determinants of health (N = 22)(Brazil). (DOCX)

S4 Table Studies on the effects of the Bolsa Familia Programme on health outcomes (N = 59) (Brazil). (DOCX)

S5 Table Studies on the effects of the *Bono de Desarollo Humano* on socioeconomic determinants of health or health outcomes (N = 15) (Ecuador). (DOCX)

S6 Table Studies on the effects of the Continuous cash benefit on socioeconomic determinants of health or health outcomes (N = 5)(Brazil). (DOCX)

S1 File PRISMA P Checklist. (DOCX)

S2 File Acknowledgment. (DOCX)

## Abbrevations

LA: Latin America
OSF: Open Science Framework
UN: United Nations
ECLAC: Economic Commission for Latin America and the Caribbean (also known as CEPAL)
PAHO: Pan American Health Organization
OECD: Inter-American Development Bank, World Bank and Organisation for Economic Co-operation and Development
TIDieR-PHP: Template for Intervention Description and Replication for population health and policy
SWiM: Synthesis Without Meta-analysis
SDH: Socioeconomic determinants of health
BFP: Programa Bolsa Familia/ Bolsa Familia Program
BPC: Beneficio de Prestacao Continuada/ Continuous Cash Benefit
BDH: Bono de Desarrolo Humano/ Human Development Grant
SUIBE: Sistema Único de Informações de Benefícios
PNAD: Pesquisa Nacional por Amostra de Domicílios/ Brazil’s National Household Sample Survey
INSS: Instituto Nacional do Seguro Social/ Brazil’s National Institute of Social Security
BRL: Brazilian reais
ICF: International Classification of Functioning, Disability and Health
ENEMDU: Encuesta Nacional de Empleo, Desempleo y Subempleo
RDD: regression discontinuity design
