# Supplementary material for: Implementation and effects of social protection programs for children, older adults, and people with disabilities in Brazil and Ecuador: A scoping review
Source: PLOS Glob Public Health. 2025 Oct 29;5(10):e0005281. doi: 10.1371/journal.pgph.0005281 (PMC12571297; doi:10.1371/journal.pgph.0005281)
Supplement: S1 Table — (DOCX) [file pgph.0005281.s001.docx]

## S1 Table. Eligibility criteria for selecting papers.

|  | **Social protection programmes for children experiencing poverty** | | **Social protection programmes for elders and people with disabilities experiencing poverty** | |
| --- | --- | --- | --- | --- |
|  | *Inclusion* | *Exclusion* | *Inclusion* | *Exclusion* |
| *Population* | Children and adolescents aged 17 years or younger experiencing poverty as well as their caregivers.  Studies conducted at national or subnational level | Evaluations not performed specifically or partially on children and adolescents or caregivers. | Elders aged 65 years or over experiencing poverty, individuals from all ages with long-term disabilities that can prevent them from working, as well as their caregivers.  Studies conducted at national or subnational level | Evaluations not performed specifically or partially on elders and people with disabilities or their caregivers |
| *Intervention* | Bolsa Familia Programme  Bono de Desarrolo Humano  Bono 1000 días |  | Beneficio de Prestacao Continuada (BPC)  Pensión Mis Mejores Años/Pensión para adultos mayores  Pensión Toda una Vida/Pensión para personas con discapacidad  Bono Joaquín Gallegos Lara |  |
| *Implementation* | Policy implementation: quality, speed, coverage, and mechanisms of control  Effects on social determinants of health (e.g., social position, economic and social inequality, income, education, employment or occupation, material and household circumstances) and health (e.g., morbidity and mortality). |  | Policy implementation: quality, speed, coverage, and mechanisms of control  Effects on social determinants of health (e.g., social position, economic and social inequality, income, education, employment or occupation, material and household circumstances) and health (e.g., morbidity and mortality). |  |
| *Effects* |  | Effect evaluation studies without a comparison group.  Studies comparing any of the seven selected programmes with other programmes not included in this scoping review | No benefit, alternative benefit or before and after. | Effect evaluation studies without a comparison group.  Studies comparing any of the seven selected programmes with other programmes not included in this scoping review |
