# Supplementary material for: Implementation and effects of social protection programs for children, older adults, and people with disabilities in Brazil and Ecuador: A scoping review
Source: PLOS Glob Public Health. 2025 Oct 29;5(10):e0005281. doi: 10.1371/journal.pgph.0005281 (PMC12571297; doi:10.1371/journal.pgph.0005281)
Supplement: S2 Table — (DOCX) [file pgph.0005281.s002.docx]

**S2 Table.** PIIE (Population, Intervention, Implementation and Effects) search terms.

| **PIIE** | **Search terms** |
| --- | --- |
| Population | English:  children OR elderly OR older OR retired OR disabled OR orphan OR catastrophic diseases OR rare diseases  Portuguese:  Crianca OR idoso OR aposentado OR aposentada OR orfa OR incapacidade OR doenca incapacitante OR doenca rara  Spanish:  Nino OR nina OR idoso OR aposentado OR aposentada OR orfa OR incapacidade OR enfermedad discapacitante OR enfermedad raras |
| Intervention | English:  “Bolsa Familia” OR “Continuous Benefit Programme” OR Human Development benefit OR Human Development allowance OR Programme First 1000 days OR Elder benefit OR disability benefit OR elder benefit OR disability pension OR disability allowance OR cash for elders OR cash for children OR cash for poor OR cash for disabilities  Portuguese:  “Beneficio de Prestacao Continuada” OR pensao Melhores Anos OR pensao idosos OR pensao para desenvolvimento humano OR beneficio para desenvolvimento humano OR programa para desenvolvimento humano OR pensao 1000 dias OR beneficio 1000 dias OR programa 1000 dias OR pensao idosos OR beneficio idosos OR pensao incapacidades OR beneficio incapacidades  Spanish:  “Pension of Best Ages” OR “Pensión Mis Mejores Años” OR “Pensión para adultos mayores” OR “Pensión para personas con discapacidad” OR “Pensión Toda una Vida” OR “Joaquín Gallegos Lara” OR Bono Mejores Anos OR Pension mejores anos OR Bono de Desarrollo Humano OR Bono 1000 días OR Bono toda una vida OR Bono para discapacidad |
| Implementation | No search will be performed for it. Instead, we will use the defined selection criteria to select eligible studies. |
| Effects | English/Spanish/Portuguese:  (implement* OR income OR renda OR econom* OR “poverty” OR develop* OR inequalit* OR inequi* OR desenvolvim* OR desarollo OR empleo OR employ* OR desempreg* OR empreg* OR “malnutrition” OR “nutrition” OR ((“food” OR comid* OR aliment*) AND (security OR availability OR access* OR acess*)) OR educat* OR educ* OR mortality OR mortalid* OR death OR morte OR morbid* OR diseas* OR disabili* OR illness* OR “ill” OR enfermed* OR health OR salud OR saúde OR vaccin* OR vacin* OR imuniza* OR “birth weight” OR ((treat* OR tratam* OR medic*) AND (outcom* OR desfech* OR dropout)) |
