# Supplementary material for: Implementation and effects of social protection programs for children, older adults, and people with disabilities in Brazil and Ecuador: A scoping review
Source: PLOS Glob Public Health. 2025 Oct 29;5(10):e0005281. doi: 10.1371/journal.pgph.0005281 (PMC12571297; doi:10.1371/journal.pgph.0005281)
Supplement: S3 Table — (DOCX) [file pgph.0005281.s003.docx]

**S3 Table.** Studies on the effects of the **Bolsa Familia Programme** on socioeconomic determinants of health (N=22)(Brazil).

| **Author/Year (et al)** | **Study setting and dataset** | **Study design & methods of analysis** | **Study population** | **Sample size** | **Definition of the exposure and comparison groups** | **Outcome(s)** | **Overall effect direction** |
| --- | --- | --- | --- | --- | --- | --- | --- |
| ***Effects on socio-determinants of health*** | | | | | | | |
| Alves, 2013  [51] | Study using primary data from Brazil-Municipio de Silva Jardim-RJ, collected in 2011-2012. | Qualitative, Individual, case study employing participant observation, documentary research, and semi-structured interviews. Data collection was conducted using a specific instrument tailored for each family group, allowing an in-depth exploration of household dynamics and processes of social inclusion and exclusion. | Individuals in Families living in extreme poverty, including 25 individuals from beneficiary families and 6 individuals from former beneficiary families of the Bolsa Família Program (BFP). | 31 | N/A | Social vulnerabilities | Benefit |
| Amaral and Goncalves, 2015  [34] | Study using data from the 2010 Brazilian Demographic Census | Individual,  cross-sectional study with descriptive analyses and binary logistic regression models, adjusting for household, maternal, and child characteristics. Data were stratified by three income thresholds: up to R$70, R$140, and R$280 per capita. | Children aged 7–14 years | 167,797 | Exposed group: Children from households receiving BFP benefits.   Comparison group: Children from households not receiving BFP benefits. | School enrolment | Benefit |
| Amaral and Monteiro, 2013  [35] | Study using data from the 2005 and 2009 Impact Evaluation of the Bolsa Família Program (AIBF), conducted by the Ministry of Social Development (MDS). The data included a nationally representative sample from three regions of Brazil: North/Central-West, Northeast, and South/Southeast. | Individual, panel data analysis using logistic regression models to estimate the association between household, maternal, and child characteristics and school dropout rates. | Children aged 7–14 years from households with a maximum per capita income of R$200 (2005) and R$280 (2009), including households receiving Bolsa Família benefits, and  households that reported never receiving any type of social benefit, regardless of enrolment in public programs. | 2005: 9,232 2009: 8,437 | Exposed group: Children from households receiving Bolsa Família benefits.  Comparison group: Children from households that reported never receiving any type of social benefit, regardless of enrolment in public programs. | School  dropout rates | Benefit |
| Aransiola & Justus, 2020  [48] | Study using data from the National Household Sample Survey (PNAD) and administrative records from the Ministry of Social Development and Ministry of Labour and Employment, covering child labour trends in Brazil between 2000 and 2014. | Area level, longitudinal study using aggregated data from Brazilian states to examine the evolution of child labour rates and the effectiveness of governmental countermeasures, including Bolsa Família (BFP) and Labour Inspections. | Children aged 5–15 years, with a focus on those engaged in child labour across urban and rural settings in Brazil. | 26 | Exposed group: States or regions with a higher incidence of child labour and stronger exposure to Bolsa Família benefits and/or labour inspections.  Comparison group: States or regions with lower coverage of Bolsa Família and/or fewer labour inspections, used as a baseline for assessing policy impact. | Child labour rates | No difference or contradictory effects |
| Barbosa & Corseuil, 2014  [52] | The study used data from the 2006  National Household Sample Survey (PNAD). | Household, cross-sectional study with Fuzzy Regression Discontinuity Design (RDD) to evaluate the impact of Bolsa Família on labour market outcomes, specifically the allocation of labour between formal and informal sectors. | Households with children around the eligibility threshold for Bolsa Família benefits in 2006, focusing on those with the youngest child near the age limit of 16 years. | 145,547 households and 410, 241 individuals. | Exposed group: Households eligible for Bolsa Família benefits due to having a youngest child aged below 16 years on December 31, 2005.  Comparison group: Households ineligible for Bolsa Família benefits due to having a youngest child aged 16 years or older on the same date. | Labour allocation between formal and informal sectors | No difference or contradictory effects |
| Brauw et al 2015  [36] | The study utilized longitudinal household-level data collected in 2005 and 2009 as part of the Bolsa Família Impact Evaluation (AIBF). Data include over 11,000 households across Brazil, specifically designed to evaluate Bolsa Família. | Household, longitudinal study using propensity-score-weighted regression models to assess program impacts on school participation, grade progression, and dropout rates, disaggregated by sex, age, and location. | Children aged 6–17 years | 2,828 households receiving Bolsa Família transfers in 2009  and 2,586 households not receiving Bolsa Família transfers in 2009. | Exposed group: Households receiving Bolsa Família transfers in 2009, registered in Cadastro Único in 2005 but not receiving Bolsa Família or similar benefits in 2005.  Comparison group: Households registered in Cadastro Único in 2005 but not receiving Bolsa Família or similar benefits in both 2005 and 2009. | School enrolment, dropout rates, grade progression, and grade repetition | No difference or contradictory effects |
| Melo & Duarte 2010  [37] | Study focused on rural family farming households in the Northeast region of Brazil, specifically in the states of Pernambuco, Ceará, Sergipe, and Paraíba. It used both primary data from the Dom Helder Câmara project and secondary data from the 2005 PNAD (National Household Survey). | Household, cross-sectional study using propensity score matching techniques (nearest neighbour, kernel, and stratification) to evaluate the impact of Bolsa Família on school attendance among children and adolescents. | Children aged 5–14 years living in rural family farming households, | 745 children from the Dom Helder Câmara project and 375 children from the PNAD dataset. | Exposed group: Children living in rural family farming households receiving Bolsa Família benefits.  Comparison group 1 (Primary data): Children from rural family farming households not receiving Bolsa Família benefits. Comparison group 2 (PNAD data): Children from rural family farming households not receiving any public cash transfers, with a per capita income below R$200. | School attendance of children | No difference or contradictory effects |
| Costa et al 2020  [49] | The study used data from the 2010 Brazilian Demographic Census, focusing on child labour and the impacts of the Bolsa Família Program (PBF) and the Child Labour Eradication Program (PETI). | Individual, cross-sectional study using Propensity Score Matching (PSM) to estimate the causal effects of the programs on child labour, both in terms of the probability of labour (extensive margin) and the weekly hours worked (intensive margin). The analysis was stratified by regions to account for heterogeneous effects. | Children aged 10–15 years in households identified as beneficiaries of Bolsa Família (PBF) or PETI programs, as well as non-beneficiary households. | 4,088,613 children for the PBF analysis and 192,333 children for the PETI analysis. | Exposed group: Households benefiting from Bolsa Família or PETI, based on their responses to income-related questions in the Census.  Comparison group: Households not receiving either Bolsa Família or PETI benefits. | Child labour | No difference or contradictory effects |
| Cruz & Ziegelhöfer 2014  [53] | The study utilized data from the Brazilian Household Expenditure Survey (POF) and administrative data to evaluate household spending patterns across Brazil,  2008-2009. | Household, cross-sectional study using fuzzy Regression Discontinuity Design (RDD) using income and the number of children as forcing variables to evaluate the impact of Bolsa Família on household expenditure decisions, focusing on private investments in child human capital, particularly in nutrition, health, and education.  Results were analysed against Engel curves to assess whether the program's effects extended beyond a pure income effect. | Households with income per capita near the Bolsa Família eligibility threshold, including both beneficiaries and non-beneficiaries, with an emphasis on families with children. | 55,976 households | Exposed group: Households receiving Bolsa Família benefits based on income eligibility and the number of children.  Comparison group: Households with similar characteristics near the eligibility threshold but not receiving Bolsa Família benefits. | Spending on food and education | Benefit |
| Draeger 2021  [38] | The study utilized data from the 2006 and 2009 PNAD (National Household Survey) across all Brazilian regions. | Individual, cross-sectional study using Difference-in-Differences (DiD) approach and a classifier method to estimate both Intent-to-Treat (ITT) and Average Treatment Effects on the Treated (ATT) for school attendance among adolescents. Heterogeneous effects were analysed by gender and region.  The focus was on the 2008 Bolsa Família program expansion, which included adolescents aged 16–17. | Adolescents aged 15–16 years in the lowest income quintile | 1,336 adolescents (2006) | Exposed group: 16-year-olds from households receiving Bolsa Família benefits under the program expansion.  Comparison group: 15-year-olds from similarly low-income households not receiving Bolsa Família benefits. | School attendance rates for 16 year-olds | No difference or contradictory effects |
| Fitz & League 2019  [39] | The study utilized data from the 2005 and 2009 Bolsa Família Impact Evaluation (AIBF), covering over 15,000 households in 2005 and 11,000 in the 2009 follow-up survey. The analysis integrates these survey data with municipal-level rainfall data from the Terrestrial Air Temperature and Precipitation: 1900-2014 Gridded Monthly Time Series dataset. | Household, longitudinal study using  two identification strategies: (1) Propensity Score Weighting (PSW) using only 2009 data, and (2) Panel Fixed Effects Estimation using both 2005 and 2009 data. The rainfall shocks are used as an exogenous income shock variable to analyse their effect on schooling and child labour. Interaction terms assess whether Bolsa Família mitigates the adverse effects of these shocks. | School-aged children (6–17 years old) | 2,828 households receiving Bolsa Família in 2009 (treatment group) 2,586 households not receiving Bolsa Família in 2009 (comparison group) | Exposed group: Households that registered for Bolsa Família in Cadastro Único and received benefits in 2009 but were not recipients in 2005.  Comparison group: Households that were registered in Cadastro Único but did not receive Bolsa Família in either 2005 or 2009. | School attendance and child labour | No difference or contradictory effects |
| Gonçalves et al 2017  [40] | The study used data from the 2010 Brazilian Census | Individual, cross-sectional study using binary logistic regression models to estimate the association between Bolsa Família participation and age-grade distortion among students aged 8 to 14 years. The analysis controlled for socioeconomic characteristics, household conditions, maternal education, and regional differences. | Children aged 8–14 years | 425,782 (with income of up to R$70.00) and 871,905 (with income of up to R$140.00) | Exposed group: Children from households that self-reported as Bolsa Família beneficiaries in the 2010 Census.  Comparison group: Children from households that did not self-report as Bolsa Família beneficiaries in the 2010 Census. | Age-grade distortion | No difference or contradictory effects |
| Nascimento 2013  [47] | Study using microdata from the 2009 and 2011 National Household Sample Survey (PNAD - Pesquisa Nacional por Amostra de Domicílios). | Individual, cross-sectional study using  Propensity Score Matching (PSM) to estimate the causal impact of Bolsa Família on the likelihood of child labour. Additionally, logistic regression models were used to analyse the relationship between social transfer income and child labour outcomes. | Children aged 5 to 15 years | 2009: 16,343  2011: 10,699 | Exposed group: Children from households receiving Bolsa Família benefits in 2009 and 2011.  Comparison group: Children from households not receiving Bolsa Família in the same years. | Probability of a child working or the number of hours worked | No difference or contradictory effects |
| Nunes & Mariano 2015  [54] | Study using  Brazilian rural areas of the Northeast region data from National Household Sample Survey (PNAD - Pesquisa Nacional por Amostra de Domicílios) 2006. | Individual, cross-sectional study using Heckman’s two-step selection model and the Double Hurdle model (Cragg, 1971) to analyse both the decision to participate in the labour market and the allocation of hours worked in non-agricultural activities. | Rural households in the Northeastern region of Brazil, with a focus on the working-age population and their children (ages 11–15). | not reported | Exposed group: Households receiving Bolsa Família or other social cash transfers in 2006.  Comparison group: Households not receiving Bolsa Família or other social transfers in 2006. | Participation in non-agricultural labour among both parents and children. | Benefit |
| Peruffo & Ferreira 2017  [41] | Study using  from Nationwide Brazilian population data from various sources (e.g. National Household Sample Survey (PNAD - Pesquisa Nacional por Amostra de Domicílios 1997) and others. | Individual, cross-sectional study using  a general equilibrium modeling with heterogeneous agents, simulating a life-cycle model to estimate the effects of Bolsa Família on human capital accumulation, poverty, and inequality. The policy is modeled to replicate the actual Bolsa Família eligibility thresholds and schooling requirements, comparing steady states before and after the program’s implementation. | Brazilian population in 1997 (outcomes were assessed for children only, children's age was not reported) | Not applicable, as the study is based on a calibrated economic model rather than microdata analysis. | Exposed group: Simulated households receiving Bolsa Família, subject to income thresholds and schooling conditions, modeled to match program coverage and budget in 2012.  Comparison group: Simulated households without Bolsa Família, following the economic conditions observed in Brazil before the program’s expansion. | Child labour hours, child labour, and school attainment | Benefit |
| Ribeiro & Cacciamali 2012  [42] | Study using  data from the National Household Sample Survey (PNAD - Pesquisa Nacional por Amostra de Domicílios) in 2006 in Brazil, stratified by urban and rural areas. in 2006. | Individual, cross-sectional study using Propensity Score Matching (PSM) to estimate the causal impact of Bolsa Família on school attendance and age-grade distortion. The empirical strategy involved comparing educational indicators between beneficiary and non-beneficiary children. Difference-in-means tests were performed to assess program effects. | Children and adolescents aged 7 to 15 years | 29,886 individuals | Exposed group: Households with children aged 7–15 years that self-reported receiving Bolsa Família benefits in 2006.  Comparison group: Households with children in the same age range that did not receive Bolsa Família but had per capita income below R$300, ensuring comparability. | School attendance or age-grade distortion. | No difference or contradictory effects |
| Santos et al 2017  [43] | Study using systematic review  considering primary and secondary data sources, including PNAD (National Household Sample Survey), School Census, and standardized educational assessments (Prova Brasil, IDEB, SARESP, and others). | Systematic review conducted across multiple databases (Medline, Lilacs, EBSCO, Econstor, Science Direct, and Web of Knowledge). Studies were classified into "effect" (assessing changes over time) and "impact" (with control groups). The methodological quality of included studies was assessed using the Downs & Black checklist. | Children and adolescents aged 6 to 17 years, focusing on beneficiaries of Bolsa Família and their educational performance and school engagement. Some included studies also evaluated educational institutions with a high percentage of beneficiaries. | 12 studies | Exposed group: Children and adolescents from households benefiting from Bolsa Família, as well as schools with a high proportion of beneficiaries.  Comparison group: Children and adolescents from households not receiving Bolsa Família, and schools with a lower proportion of beneficiaries. | Dropout, attainment, absence, delay, repetition, Portuguese and mathematics proficiency (student) and grades (by school), enrolment at school, IDEB (Índice de Desenvolvimento da Educação Básica) | No difference or contradictory effects |
| Silveira et al 2018  [46] | Study using  data from the  IBGE 2010 Child Labour Census | Cross-sectional study using a quantitative ecological design, analysing aggregated data at the municipal level. A Multiple Linear Regression Model stratified by the Human Development Index (HDI) was used to assess the association between Bolsa Família resources and child labour prevalence. | Children aged 10–13 years old | 161 areas | Exposed group: Municipalities with higher Bolsa Família and Child Labour Eradication Program (PETI) allocations per capita.  Comparison group: Municipalities with lower Bolsa Família and PETI allocations per capita. | Child and adolescent labour | No difference or contradictory effects |
| Pais 2017  [45] | Study using  data from National Household Sample Survey (PNAD) in 2006. | Individual, cross-sectional study using Propensity Score Matching (PSM) to estimate the causal impact of Bolsa Família on child labour time allocation. The authors matched beneficiary and non-beneficiary households based on socioeconomic characteristics and used a nearest-neighbor matching algorithm to ensure comparability between groups. | Children and adolescents aged 5 to 17 years from households with per capita income up to R$300.00 (~US$139.53) | 21,886 individuals | Exposed group: families with children age 0 to 17 years with a net income per capita up to RS300 (excluding government transfers)  Comparison group: families suitable to receive the Bolsa Familia monetary transfer, characterized as having a similar level of economic vulnerability | Hours of child labour | Harm |
| Schwartz 2011  [44] | Study using data from Saresp 2007 (Portuguese and math test scores) from São Paulo State Secretariat of Education. Data on BFP receipt  from the SAGI, a branch of the Brazilian Ministry of Social Development in 2007 | Individual, cross-sectional study using  Independent-samples t tests, chi-square, multiple regression, and logistic regression | Students enrolled in the sixth grade in 94 public state schools in the city of Campinas in 2007 who took the Saresp 2007 test and survey. | 9,424 students | Exposed group: Students from Bolsa Família beneficiary households.  Comparison group: Students from non-beneficiary households. | Portuguese and math test scores | Harm |
| Simoes & Sabates 2014  [33] | Study using a national dataset at the school level was constructed using three different sources of administrative records from the Federal Government in Brazil: (1) the Prova Brasil 2007 dataset (2) the Bolsa Famılia dataset  and (3) the National School Census 2007 dataset in 2005 - 2007 | Area, Cross-sectional study using Adjusted analysis and 1. crossectional - OLS regression to examine marginal effect of proportion of BFP on school oucomes 2. panel data  - fixed effect regression - to estimate marginal effects of BFP school intake over time | 4th-grade students enrolled in public schools across Brazil | 23,747 schools | Exposed group: Students from Bolsa Família beneficiary households.  Comparison group: Students from non-beneficiary households in the same school system. | Test scores in Portuguese and Mathematics, dropout reduction and school progression | No difference or contradictory effects |
| Fassarella et al., 2024  [50] | Study using administrative datasets tracking children from 2005 to 2019 from Brazil - national in 2005-2019 | Individual, longitudinal/cohort study using descriptive statistics, correlation analysis, fractional logistic regression and models adjusted for individual and municipal-level variables | Children, tracked until adulthood (21-30 years old) | 11,628,308 | Exposed group: Registered as dependents in BFP Payrolls in 2005  Comparison group: Individuals never registered as BFP beneficiaries until 2019 | Social mobility, employment, emancipation from social programs | Benefit |
