# Supplementary material for: Implementation and effects of social protection programs for children, older adults, and people with disabilities in Brazil and Ecuador: A scoping review
Source: PLOS Glob Public Health. 2025 Oct 29;5(10):e0005281. doi: 10.1371/journal.pgph.0005281 (PMC12571297; doi:10.1371/journal.pgph.0005281)
Supplement: S4 Table — (DOCX) [file pgph.0005281.s004.docx]

**S4 Table.** Studies on the effects of the **Bolsa Familia Programme** on health outcomes (N=59) (Brazil).

| **Author/Year (et al)** | **Study setting and dataset** | **Study design & methods of analysis** | **Study population** | **Sample size** | **Definition of the exposure and comparison groups** | **Outcome(s)** | **Overall effect direction** |
| --- | --- | --- | --- | --- | --- | --- | --- |
| ***Effects on health*** | | | | | | | |
| Alves et al., 2023  [87] | Study using the 100 Million Brazilian Cohort, a longitudinal administrative dataset that links Secondary socioeconomic data from Cadastro Unico with national health records (SINASC - Live Birth Information System and SIM - Mortality Information System) in 2004-2015 | Individual, cross-sectional nested within a longitudinal cohort analysis using Propensity scores and Propensity score kernel weighting, controlling for sociodemographic and economic confounders. Additionally, stratified analyses are conducted based on race, urban or rural residence, municipal human development index (MHDI), and municipal primary health care coverage. | Girls and women aged 10 to 49 years, who had at least 1 live birth between 2004 and 2015, recorded in the 100 Million Brazilian Cohort. | 6,677,273 women | Exposed group: all women with records of live births who started receiving BFP before or during pregnancy and did not stop receiving the benefit until childbirth or before death.  Comparison group: Women who had not received the benefit at any time or until delivery were considered not exposed. | Maternal mortality | Benefit |
| Andrade et al., 2013  [58] | Study using the 2005 Bolsa Família Impact Evaluation Research. | Individual, cross-sectional study using Propensity Score Matching (PSM) to estimate the causal impact of Bolsa Família on child nutritional status. The methodology controls for selection bias by comparing nutritional indicators (height-for-age and BMI-for-age) between beneficiaries and non-beneficiaries, using a Nearest Neighbor Matching approach. | children aged between 6 and 60 months living in low-income households (per capita income up to R$200) | 15,240 households | Exposed group: Children aged 6–60 months from households receiving Bolsa Família benefits.  Comparison group: Children aged 6–60 months from non-beneficiary households that met eligibility criteria (income below R$200 per capita) but had never received Bolsa Família or other public transfers. | Malnutrition and extreme malnutrition | No difference or contradictory effects |
| Andrade et al., 2018  [106] | Study using data  from the Notifiable Diseases Information System (SINAN), the Social Information Matrix (MIS) of the Ministry of Social Development, and the 2000 and 2010 National Census (IBGE). | Area, longitudinal ecological design study using negative binomial regression models for panel data with fixed effects, controlling for Family Health Program (FHP) coverage and various sociodemographic covariates such as income inequality (Gini Index), illiteracy rate, unemployment, and household overcrowding. It examined the impact of Bolsa Família (BFP) coverage on the new case detection rate (NCDR) of leprosy in children under 15 years old, from 2004 to 2015. | Children under 15 years old living in 1,120 municipalities with high leprosy endemicity. | 1,120  municipalities | Exposed group: Municipalities with higher Bolsa Família coverage, categorized into tertiles (low, intermediate, and high coverage).  Comparison group: Municipalities with lower Bolsa Família coverage, using the lowest tertile as the reference group. | New case detection rate (NCDR) of leprosy | Benefit |
| Andrade et al., 2013  [99] | Study using  data from the 2005 Bolsa Família Impact Evaluation Survey. | Individual, cross-sectional study using Propensity Score Matching (PSM) to estimate the impact of Bolsa Família on child immunization rates. The authors use Nearest Neighbor Matching to compare beneficiary and non-beneficiary households while controlling for selection bias. The analysis is stratified by region (Northeast, North/Central-West, and Southeast/South) and child age group (0–3 years and 4–6 years). The analysis focuses on the impact of Bolsa Família (BFP) on child immunization rates across different Brazilian regions. | Children aged 0 to 6 years from low-income households (per capita income ≤ R$200), eligible for Bolsa Família benefits. | 8,709 children | Exposed group: Children aged 0 to 6 years from households receiving Bolsa Família benefits in 2005.  Comparison group: Children from non-beneficiary households that were eligible for Bolsa Família but never received any cash transfer. | Immunization of children | No difference or contradictory effects |
| Barcelos et al., 2017  [108] | Study using data from the 2012 external assessment of the PMAQ (Program for Improving Access and Quality of Primary Care), which evaluated Basic Health Units (BHU) across all five regions of Brazil. | Individual, cross-sectional study using Poisson regression models with robust variance to analyse associations between socioeconomic, demographic, and health service organization variables and the quality of cervical cancer screening. Three quality indicators were assessed: lack of access (never had a Pap smear), late examination (Pap smear performed more than 36 months ago), and lack of guidance (no information on the importance of screening). | Women aged 25 to 64 years who accessed Basic Health Units (BHU) participating in the PMAQ. | 35,844 Women | Exposed group: Women receiving Bolsa Família benefits.  Comparison group: Women not receiving Bolsa Família benefits. | Quality of cervical cancer screening | Benefit |
| Baptista, 2019  [59] | Study using  data from the "Prevalence and Determinants of Anemia in Women and Children in the State of Maranhão" study. The data includes socioeconomic and demographic questionnaires, a food frequency questionnaire (FFQ), and biochemical hemoglobin measurements collected from mothers and children in 2010 | Individual, cross-sectional study using statistical modeling to examine the relationship between dietary iron intake, socioeconomic conditions, and anemia prevalence. The Bolsa Família Program (BFP) is evaluated as a potential protective factor against anemia. | Women aged 15 to 49 years and children under five years old | 779 women and 966 children | Exposed group: Women and children from households receiving Bolsa Família benefits.  Comparison group: Women and children from non-beneficiary households. | Anemia prevalence | Benefit |
| Bernal et al., 2019  [109] | Study using data from the 2013 National Health Survey (Pesquisa Nacional de Saúde - PNS), a nationwide household survey conducted by the Brazilian Institute of Geography and Statistics (IBGE).  The study also included laboratory data collected in a subsample of the survey (PNS Laboratory Module (2014–2015). | Individual, cross-sectional study using descriptive and inferential statistics, including Pearson’s chi-square test to compare the prevalence of NCD indicators between BFP beneficiaries and non-beneficiaries. Prevalence ratios (PR) were adjusted for age and other covariates. | Women in reproductive age (aged 18 to 49 years) | 3,131 women | Exposed group: Women aged 18 to 49 years from households receiving Bolsa Família in July 2013.  Comparison group: Women of the same age group who did not receive Bolsa Família. | Noncommunicable disease (NCD) | Harm |
| Boi 2020  [89] | Study using data from the Brazilian Ministry of Health (DATASUS), the Ministry of Social Development (MDS), IBGE (Census data), and the Municipal Human Development Index (M-HDI) database covering the period from 2008 to 2018. | Area, longitudinal panel data approach, using fixed-effects ordinary least squares (OLS) regression models at the municipal level. The econometric models control for demographic, social, economic, and policy variables, and the study includes an interaction term between BFP coverage and FHS coverage to assess potential synergies. | All municipalities in the Northeast region of Brazil, focusing on women of reproductive age (15–49 years old) and children under 5 years old. | 1,572 municipalities | Exposed group: Municipalities with higher Bolsa Família coverage (measured as the proportion of beneficiary households).  Comparison group: Municipalities with lower Bolsa Família coverage. | Infant, child, and maternal mortality rates. | Benefit |
| Camelo et al., 2009  [60] | Study using  data from the 2006 National Demographic and Health Survey (Pesquisa Nacional de Demografia e Saúde - PNDS 2006), conducted by the Brazilian Health Ministry. | Household, cross-sectional study using Propensity Score Matching (PSM) to estimate the impact of Bolsa Família on food security, children's nutritional status, and child mortality. The Brazilian Food Insecurity Scale (EBIA) was used to measure household food security, while anthropometric indicators (height-for-age, weight-for-age, weight-for-height, and BMI-for-age) were assessed using WHO 2006 standards. Child mortality was evaluated at the household level. | Households with children aged 0 to 6 years from low-income families | 2,794 households were included in the food security assessment. 2,542 children were analysed for anthropometric indicators. 2,454 children were included in the child mortality analysis. | Exposed group: Households receiving Bolsa Família benefits at the time of the survey.  Comparison group: Households eligible for Bolsa Família but not receiving benefits. | Household food security; child mortality; nutritional status | No difference or contradictory effects |
| Costa Silva 2020  [57] | Study using data from the 2008–2009 Household Budget Survey (Pesquisa de Orçamentos Familiares - POF), conducted by the Brazilian Institute of Geography and Statistics (IBGE). | Individual, case control study using quantile regression models to estimate the relationship between BFP participation and Body Mass Index (BMI), controlling for caloric intake and other factors. A Quadratic Almost Ideal Demand System (QUAIDS) was also used to analyse food demand at the household level, with adjustments to estimate individual consumption based on household data. The analysis incorporated Propensity Score Matching (PSM) to reduce selection bias in evaluating the effects of BFP. | Children from 2 to 9 years old | 6,129 children with resident mothers in the household. For broader household-level analysis, the final sample included: 126,375 individuals in 2002/2003 and 142,683 in 2008/2009, corresponding to a population of 123,352,543 and 146,499,989 individuals, respectively | Exposed group: Children aged 2 to 9 years in households receiving Bolsa Família benefits in 2008–2009.  Comparison group: Children in non-beneficiary households meeting Bolsa Família eligibility criteria. | Nutritional status and demand for food | No difference or contradictory effects |
| Oliveira et al., 2011  [62] | Study using primary data from a survey conducted in Paula Cândido, a small municipality in the Zona da Mata region of Minas Gerais, Brazil, from September to November 2007. | Individual, Cross-sectional study using  descriptive and inferential statistical methods, such as Pearson’s chi-square test, Student’s t-test, and Mann-Whitney test, to compare nutritional outcomes (anthropometry and hemoglobin levels) between beneficiaries and non-beneficiaries of Bolsa Família. | Children aged 6 to 84 months, registered in the Bolsa Família Program. | 446 children | Exposed group: Children aged 6 to 84 months from households receiving Bolsa Família benefits.  Comparison group: Children aged 6 to 84 months from registered households not receiving Bolsa Família benefits, despite meeting eligibility criteria. | Nutritional status and anemia, short stature and obesity. | No difference or contradictory effects |
| Oliveira et al., 2022  [63] | Study using primary data from a survey conducted in seven municipalities in the interior of Paraíba, Brazil, in 2018, targeting children under five years of age served by the Family Health Strategy (FHS). | Individual, cross-sectional study analysing data through decision tree models (CHAID algorithm) to identify associations between stunting, overweight/obesity, and maternal/child factors. Statistical significance was determined with chi-square tests corrected by Bonferroni adjustments. | Children aged 0 to 59 months | 479 children | Exposed group: Children from households receiving Bolsa Família benefits.  Comparison group: Children from households not receiving Bolsa Família, despite being served by the FHS. | Stunting and overweight/obesity | No difference or contradictory effects |
| Calvasina et al., 2018  [100] | Study using primary data from a survey conducted in Fortaleza, Ceará, Brazil, during 2016. | Individual, cross-sectional study using descriptive statistics and multiple logistic regression models to identify factors associated with the prevalence of dental caries, considering covariates such as socioeconomic status, oral health behaviors, and caregiver characteristics. The main outcome variable was the presence of dental caries, measured using the dmft index (decayed, missing, and filled teeth). | Five-year-old children and her/his parent/guardian, whose families are registered in the national unified social system registry (CADUNICO) | 230 pairs of caregivers/children | Exposed group: Five-year-old children from families receiving Bolsa Família benefits.  Comparison group: Five-year-old children from families not yet receiving Bolsa Família benefits, despite being eligible. | Oral health and dental caries. | Benefit |
| Carmo et al., 2016  [64] | Study using primary data from a survey conducted in municipal schools of Belo Horizonte, Minas Gerais, Brazil, in 2009. | Individual, cross-sectional study using statistical analyses included Mann-Whitney tests, chi-square tests, and Poisson regression models with robust variance, with a significance level of 5%. Food frequency was assessed through a simplified Food Frequency Questionnaire (FFQ), and anthropometric indices were calculated based on WHO growth standards. | Children enrolled in the fourth grade of municipal schools, aged 8.6–11.9 years, with data obtained from both students and their mothers/guardians. | 319 students | Exposed group: Children from families receiving Bolsa Família benefits.  Comparison group: Children from families not receiving Bolsa Família benefits, despite attending the same municipal schools. | Dietary patterns and nutritional status | No difference or contradictory effects |
| Dal Bom et al., 2019  [61] | Study using primary data from a survey  from Cruzeiro do Sul city in july 2016 to June 2017 - The baseline data were obtained between July 1, 2015 and June 30, 2016 in the Women’s and Children’s Hospital of Jurua´ Valley | Individual, longitudinal/Cohort study using  a hierarchical conceptual framework for multiple linear regression models to evaluate associations with linear growth (LAZ) and weight-for-age (BAZ). Factors were analysed at distal, intermediate, and proximal levels. | Children aged 10–15 months | 772 children were evaluated during the follow-up, representing 62% of the eligible participants from the initial cohort of 1,551 live births. | Exposed group: Children from households receiving the Bolsa Família Program (BFP).  Comparison group: Children from households not receiving Bolsa Família benefits, despite being part of the study cohort. | Linear growth and weight attained | No difference or contradictory effects |
| Dallazen et al., 2022  [66] | Study using primary data from a survey conducted in 48 of the poorest municipalities in the South Region of Brazil, between January and June 2015. | Individual level, Cross-sectional study using data collection included socioeconomic questionnaires answered by legal guardians and venous blood samples from children to determine plasma retinol concentrations using High-Performance Liquid Chromatography (HPLC). A Poisson regression model with robust variance was used to identify risk factors associated with VAD. | Children aged 12–59 months | 1,503 children | Exposed group: Children from households receiving Bolsa Família benefits.  Comparison group: Children from households not receiving Bolsa Família benefits. | Prevalence of vitamin A deficiency (VAD) | Harm |
| Facchini et al., 2014  [67] | Study using primary data from a survey  conducted in urban households with children under seven years old in the Northeast and South regions of Brazil, in areas covered by primary health care units. | Household,  cross-sectional study using Poisson regression models with robust variance for adjusted analysis, considering factors associated with moderate or severe food insecurity. | Households with children under seven years old living in urban areas of the Northeast and South regions of Brazil. Families were selected from census tracts covered by traditional and Family Health Strategy (ESF) units. | 5,419 households Northeast Region and 5,081 in the South Region | Exposed group: Households that received Bolsa Família benefits.  Comparison group: Households that did not receive Bolsa Família benefits, despite some potentially being eligible. | Food insecurity | No difference or contradictory effects |
| Ford et al., 2020  [68] | Study using primary data from a survey conducted in Acrelândia, a municipality in the Western Brazilian Amazon, using data from a population-based longitudinal study. Baseline data was collected in 2007, with follow-up in 2009. | Individual level, longitudinal/Cohort study using Poisson regression models with robust variance to assess associations between Bolsa Família participation and health/nutritional outcomes over time. The study analysed school enrolment, vaccination coverage, height and body mass index (BMI) for age z-scores, and biomarkers of micronutrient deficiencies (iron and vitamin A) among Bolsa Família participants and nonparticipants. | Children aged 10 years and younger | 1063 children in the baseline (2007). By 2009, 805 children remained in the anthropometric analysis, and 402 children participated in the biomarker analysis. | Exposed group: Children from families receiving Bolsa Família benefits.  Comparison group: Children from families who did not receive Bolsa Família benefits. | Nutritional status, school enrolment and vaccination coverage | No difference or contradictory effects |
| Labrecque et al., 2018  [65] | The study used data from the 2004 Pelotas Birth Cohort Study, conducted in Pelotas, Brazil. This is a longitudinal birth cohort that followed children from birth to 24 months, linking data with Brazil’s Bolsa Família payment database to analyse the effects of conditional cash transfers on early childhood growth indicators. | Individual level, Longitudinal/Cohort study using Targeted Maximum Likelihood Estimation (TMLE), a doubly robust and semiparametric approach to estimate causal effects, reducing the risk of residual confounding. The study categorized children into three groups based on total Bolsa Família payments received over 24 months: No Bolsa Família (R$0 received) Low Bolsa Família (≤R$1000 received) High Bolsa Família (>R$1000 received) The primary outcomes were length-for-age z-score (LAZ) and weight-for-age z-score (WAZ) at 24 months, using WHO growth standards. | children up to 24 months  from low-income families (≤R$100 per capita) participating in the 2004 Pelotas Birth Cohort Study. | 1,703 children | Exposed group: Children from households receiving Bolsa Família benefits.  Comparison group: Children from households eligible for Bolsa Família but not receiving benefits. | Child nutritional status as measured by length-forage z-score (LAZ) and weight-for-age z-score (WAZ) | Harm |
| Oliveira DS et al., 2017  [70] | Study using primary data from a survey with Families from the urban area of the municipality of Santo Antonio de Jesus, Bahia, in Dec 2011 to May 2012.  The Brazilian Food Insecurity Scale (EBIA) was used for classification. | Families, Cross-sectional study using Unadjusted and Descriptive analyses.  The chi-square test was applied for statistical associations, and proportions were used for descriptive analysis. | Urban households in a low-income neighborhood of Santo Antônio de Jesus, focusing on families with children and socioeconomic vulnerability. | 445 families (1 individual was selected per family to respond to the interview) | Exposed group: Families receiving Bolsa Família benefits.  Comparison group: Families not receiving Bolsa Família benefits, despite living in similar socioeconomic conditions. | Food insecurity | Harm |
| Oliveira et al., 2022  [63] | Study using primary data from a survey with children under 5 living in urban areas of 7 municipalities (30,000 to 149,999 inhabitants) from Paraíba, covered by the Family Health Strategy Program (ESF), 2018. | Individual, cross-sectional study using bivariate analysis, decision tree model (CHAID algorithm). The excess weight analysis estimates were adjusted by the familiy SES and mother's heigh. | Children aged 0-59 months, users of the ESF | 469 children | Exposed group: Children from households receiving Bolsa Família benefits.  Comparison group: Children from households not receiving Bolsa Família benefits, despite being part of the same urban areas served by the FHS. | Stunting and overweight/obesity | No difference or contradictory effects |
| Oliveira LJC et al., 2013  [101] | Study using primary data from a survey with Children aged 8-12, enrolled  in schools in the urban area of Pelotas, RS, Southern  Brazil, 2010. | Individual, cross-sectional study using Adjusted analysis and Poisson multivariate regression models with robust variance analysis were used to estimate the association of BFP with dental caries (prevalence and severity). Stepwise procedue was used to select the variables for the models, with backward elimination. Prevalence ratios of dental caries and 95% CI were estimated (adjusted for age and presence of plaque). Rate ratios (RR) were estimated for the severity of dental caries (adjusted for maternal schooling, age and presence of plaque). | Children aged 8-12 years old. | 1,107 children | Exposed group: Schoolchildren from households receiving Bolsa Família.  Comparison group: Schoolchildren from non-beneficiary households, including those attending public and private schools. | Dental caries in permanent teeth and severity of caries in mixed dentition | Harm |
| Olson et al., 2019  [107] | Study using  PNAD 2005-2009, 2011-2013, nationally representative household survey in 2005-2009, 2011-2013. Additionally, data from Brazil’s National Birth Registry (SINASC) was used to validate the findings. | Individual, cross-sectional study using adjusted analysis and a triple difference estimation strategy to identify an intent-to-treat estimate of the BFP. The analysis on the fertility outcomes of treated vs. non-treated was based on income eligibility, age eligibility, and timing of program implementation. Additional control variables were n of children <5y (except adolescents' children), n of family members over 5y (except adolescents' children), education, rural/urban area, state-cohort cluster for standard errors. | Adolescents girls aged 15-18y | 107,581 adolescents | Exposed group: Adolescent girls eligible for Bolsa Família benefits, defined by income level and age eligibility under the program's expansion.  Comparison group: Adolescent girls not eligible due to either higher household income or age restrictions at the time of program expansion. | Teenager fertlity | Benefit |
| Paes Souza et al., 2011  [71] | Study with children under 5 from 419 municipalities in 23 Brazilian states, data collected in 2005–2006 in vaccination campaigns promoted by the Brazilian government (Health and Nutrition Day surveys, to estimate the prevalence of anthropometric deficits in children). | Individual, cross-sectional study using adjusted analysis and Logistic regression models adjusted for Normal birth weight (≥2.5 kg), Lack of birth certificate, Family head schooling ≥5 years, Male family head, House with electricity, and Household with piped water. ORs and 95% CIs were estimated. | Children under 5 years old | 22,375 impoverished children under 5 years of age | Exposed group: Children from families receiving Bolsa Família benefits.  Comparison group: Children from non-beneficiary families, residing in similar socioeconomic conditions. | Height for age,  Weight for age and  Weight for height. | Benefit |
| Palmeira et al., 2022  [69] | Study using  2010 IBGE Census and 2017-2018 Household Budget Survey (Pesquisa de Orçamentos Familiares - POF) | Household, cross-sectional study using adjusted analysis and multinomial logistic regression models,  performed separately for rural and urban areas, adjusting for N of household members, sex, years of education of the head of the household, race/color of the head of the household, household situation (), monthly per capita family income, presence of retired individual in the household, daily access to water (only for urban area model), and an interaction term BFP x income. | Households in urban and rural areas of Northeast Brazil, particularly those in vulnerable conditions. | 17,848,855 households | Exposed group: Households receiving Bolsa Família and/or other governmental income transfer benefits.  Comparison group: Households not receiving Bolsa Família or other governmental benefits, regardless of eligibility status. | Food insecurity | No difference or contradictory effects |
| Paula et al., 2012  [73] | Study using primary data from a survey with children aged 6-10y, from a municipal school of the Sanitario Leste district, Belo Horizonte, Minhas Gerais, in a highly deprivated area, collected on August to October 2009. | Individual, cross-sectional study using descriptive analysis and hypothesis testing. The statistical methods included Kolmogorov-Smirnov test, Student’s t-test, chi-square test, and Fisher’s exact test, with a 5% significance level. Anthropometric data were analysed based on BMI-for-age and height-for-age indicators, classified using WHO growth curves. Food consumption was assessed using a qualitative dietary questionnaire. | Schoolchildren aged 6 to 10 years | 115 children | Exposed group: Schoolchildren from households receiving Bolsa Família and/or Bolsa Escola Municipal.  Comparison group: Schoolchildren from households not receiving income transfer benefits. | Weight,  height and food consumprion | No difference or contradictory effects |
| Pedraza 2021  [74] | Study using primary data from a survey  with Children <5y from families attended in the Family Health Strategy, residing in two municipalities of the metropolitan region of João Pessoa (PB, Brazil), in July to December 2014. | Individual, cross-sectional study using adjusted analysis and multiple logistic regression model, adjusted for all variables associated with the outcome (p<0.05): child characteristics (episode of diahrrea in the previous month) and socioeconomic characteristics of the family (maternal education, per capita family income, and BFP receipt). Prevalence ratios and 95% CI were estimated. | Families with children under five years old residing in urban areas covered by the Family Health Strategy (FHS) in João Pessoa's metropolitan region. | 324 children | Exposed group: Families receiving Bolsa Família benefits.  Comparison group: Families not receiving Bolsa Família benefits, despite being in similar socioeconomic conditions. | Food Insecurity | Harm |
| Pedraza and Oliveira 2021  [75] | Study using primary data from a survey  with children from families attended in the Family Health Strategy, residing in two municipalities of the metropolitan region of João Pessoa (PB, Brazil), in July to December 2014. | Individual, cross-sectional study using adjusted analysis and variables with statistically significant mean differences (t-test <0.20) for the outcomes were added to multiple linear regression models (using backward elimination procedure for variables selection for a p<0.05). BFP was not significant in descriptive analysis. | Children under 5 years old | 324 children | Exposed group: Families receiving Bolsa Família benefits.  Comparison group: Families not receiving Bolsa Família benefits, but covered by the same primary healthcare network. | Children's nutritional status | No difference or contradictory effects |
| Pedraza et al., 2013  [72] | Study using primary data from a survey from children from families enrolled in daycare centers registered in the State Secretary of Human Development of the Government of Paraíba, residing in 8 municipalities (João Pessoa, Campina Grande, Areia, Bayeux, Mamanguape, Itaporanga, Soledade and Umbuzeiro) from Paraíba, Brazil, in 2008. | Household, cross-sectional study using adjusted analysis and variables with statistically significant proportion differences (chi=square test <0.05) for the outcome were added to multiple regression models adjusted for total breastfeeding duration, exclusive breastfeeding duration, mother's age, mother's height, type of household (own or rented), per capita family income, and BFP receipt (yes/no). Odds ratios and 95%CIs were estimated. | Families with children aged 6 to 72 months enrolled in public daycare centers in socioeconomically vulnerable areas of Paraíba. | 332 children | Exposed group: Families receiving Bolsa Família benefits.  Comparison group: Families not receiving Bolsa Família benefits, despite similar socioeconomic conditions. | Food Insecurity | Harm |
| Pedraza et al., 2013  [77] | Study using primary data from a survey with Children aged 12-72 months attending public day care centers of the Government of the State of Paraiba in 2009. | Individual level, Cross-sectional evaluating the growth profile and micronutrient deficiencies (vitamin A, iron, and zinc) among children. Anthropometric measurements and biochemical analyses (serum retinol, zinc, and hemoglobin levels) were conducted to assess nutritional deficiencies. Statistical analysis included bivariate and multivariate tests to identify associations. | Children aged 12-72 months | 240 children | Exposed group: Children from households receiving Bolsa Família benefits.  Comparison group: Children from households not receiving Bolsa Família benefits, despite similar socioeconomic backgrounds. | Nutritional status and  Nutrients deficiency. | No difference or contradictory effects |
| Pedraza & Gama, 2015  [78] | Study using primary data from a survey with Children <5y attending municipal day care centers in Campina Grande, PB, Brazil. in October and November 2011. | Household, Cross-sectional study using adjusted analysis and poisson regression models with robust variance was used to estimate prevalence ratios (PR) and 95% CI, adjusted for garbage destination (collected/not collected), water supply (public network/others), type of drinking water (treated or mineral/untreated), toilet in the household (individual with flush/others), household type (brick/others), n of people in the household (< 6/≥ 6), refrigerator in the household (yes/no), BFP (yes/no). | Families with children under five years old | 793 families | Exposed group: Families receiving Bolsa Família benefits.  Comparison group: Families not receiving Bolsa Família benefits, despite living in similar socioeconomic conditions. | Food Insecurity | No difference or contradictory effects |
| Pescarini et al., 2020  [105] | Study using CadÚnico, the National data linked from Information System for Notifiable Diseases (SINAN) for leprosy and Bolsa Família payroll dataset using the 100 Million Brazilian Cohort from 2001-2015 | Individual, cohort study using propensity score matching and weighting based on household and individual socioeconomic covariates. Logistic regression to assess ATE on the treated of receipt of PBF benefits on leprosy treatment adherence and cure | Subgroup of children under 15 years of age. Newly diagnosed patients with leprosy who had started receiving PBF benefits before their leprosy diagnosis and continued receiving benefits during the entire duration of treatment. | 2,654 children <15 years | Exposed group:  Individuals (children under age 15 years) who never received PBF aid over the duration of multidrug therapy treatment were considered to be unexposed to the PBF intervention  Comparison group: Children under 15 years of age. Newly diagnosed patients with leprosy who had started receiving PBF benefits before their leprosy diagnosis and continued receiving benefits during the entire duration of treatment. | Adherence to leprosy multidrug therapy, cure in multibacillary cases | No differences |
| Wieczorkievicz et al 2017  [79] | Study was using data from the Programa Saúde na Escola (PSE) database from 2013 and the Sistema de Vigilância Alimentar e Nutricional da Criança (SISVAN-criança). | Individual, cross-sectional study using descriptive statistics and chi-square tests to assess the relationship between Bolsa Família participation and child weight and immunization status. The study included anthropometric and vaccination data collected from five municipal daycare centers (four in urban areas, one in a rural area). | Children aged 0 to 4 years and 11 months enrolled in municipal daycare centers in Mafra, Santa Catarina. | 308 children | Exposed group: Children from households receiving Bolsa Família benefits.  Comparison group: Children from households not receiving Bolsa Família benefits, despite attending the same daycare centers. | Body mass index  and Vaccination status | No difference or contradictory effects |
| Ziebold et al., 2021  [104] | Study using  2004 Pelotas Birth Cohort study database, a population-based birth cohort of children born in Pelotas, RS, from January 1 to December 31, 2004. For this study, children were followed-up from 2010 (when they were 6y) to 2015 (when they were 11y). | Individual, longitudinal/cohort study using Propensity scores matching (PSM), calculated as the probability of being a BFP beneficiary given observed covariates for the child, mother, and household. The  BFP participation covariates were selected using probit regression models and estimated covariates of the outcomes of interest using generalised linear models. The PSM was estimated according to variables strongly associated to both BFP participation and outcomes: household wealth index, maternal and child characteristics . Mental health was measured using the Strengths and Difficulties Questionnaire (SDQ), Development and Well-Being Assessment (DAWBA), and Nowick-Strickland Internal-External Scale. | Children born in 2004 in Pelotas, Brazil, who were followed up at ages 3, 12, and 24 months, as well as 4, 6, and 11 years. | 2,063 children | Exposed group: Children from households receiving Bolsa Família benefits at age 6.  Comparison group: Children from households not receiving Bolsa Família benefits at age 6, but within similar economic strata. | Mental health at age 11 | No difference or contradictory effects |
| Saldiva et al., 2010  [76] | Study using primary data from a survey conducted in João Câmara, a municipality in the semi-arid region of Rio Grande do Norte, Brazil,  collected in 2005 and 2006.. | Individual, cross-sectional study using adjusted analysis and logistic regression using binary indication of higher vs lower consumption of each food type and BFP receipt. Adjustmet was done for age (<= ou > de 24 months), head of household employed/not-employed and  worm elimination. | Children aged 6 months to 5 years. | 189 children | Exposed group: Children from households receiving Bolsa Família benefits.  Comparison group: Children from households not receiving Bolsa Família benefits, despite living in the same region. | Type of  food consumption | No difference or contradictory effects |
| Santos et al., 2021  [81] | Study conducted in Ouro Preto, Minas Gerais, Brazil, using data from the Food and Nutrition Surveillance System (SISVAN) and the Cadastro Único (CadÚnico) from 2008 to 2013. | Individual, longitudinal study using adjusted analysis and mixed regression model containing fixed and random effects, which considers a temporal sequence of two or more observations, with an unbalanced structure (measured in each individual observed at different times) and nested within the same individual. | Children aged zero to seven years old receiving BFP. | 1,353 children | Exposed group: Receiving BFP for > 180 days  Comparison group: Receiving BFP for ≤ 180 days | anthropometric index weight for age | Benefit |
| Dos Santos et al., 2020  [82] | Study using  from Two municipalities of Paraiba state: Bayeux and Cabedelo, using primary data from a household survey conducted in 2017. | Family, cross-sectional study using unadjusted and chi-squared test using Bonferroni correction and a decision tree analysis to show which factors were mostly associated with the pevalence of nutritonal insecurity. Variables analysed included socioeconomic conditions, Bolsa Família participation, maternal and child characteristics, and healthcare access. | Families with children aged 0 to 59 months living in areas covered by the Family Health Strategy (FHS) in João Pessoa’s metropolitan region. | 406 families | Exposed group: Families receiving Bolsa Família benefits.  Comparison group: Families not receiving Bolsa Família benefits, despite being covered by the same healthcare system. | Food and Nutritional Insecurity | Harm |
| Schmidt, 2016  [20] | The study was conducted in Pelotas, Brazil, using data from the 2004 Pelotas Birth Cohort, a longitudinal study that followed children from birth to six years old. | Individual, Longitudinal/Cohort study using adjusted analysis and effect was estimated using linear and Poisson regression controling for potential confounders. Potential confounders included determinants of growth and nutritional status at baseline (race/colour of the mother, child sex, maternal age, birth weight, prematurity, number of children living with the mother, maternal BMI; and at the end of follow-up (per capita income without BFP, having a partner, maternal education and work status. | Children born in Pelotas in 2004 | 4,231 children | Exposed group: Children from households receiving Bolsa Família benefits in 2010 and those who had received benefits between 2004-2010.  Comparison group: Children from households that were eligible but did not receive Bolsa Família benefits. | Score z (IMC for age) obesity | No difference or contradictory effects |
| Silva et al., 2020  [98] | The study was conducted in two Brazilian municipalities: Ribeirão Preto (São Paulo) and São Luís (Maranhão), using data from the Brazilian Ribeirão Preto and São Luís Birth Cohort Studies (BRISA). | Individual, longitudinal/Cohort study using Propensity scores and Two estimation procedures were used: pairing by propensity score by the nearest neighbor method and weighting by the inverse of the probability of exposure. The variables used for the matching are: economic class and mother’s skin color and schooling. | Children born in 2010 in Ribeirão Preto and São Luís, who were followed up between 2011 and 2013 to assess vaccination status. | 1,229 children from São Luís and 532 from Ribeirão Preto | Exposed group: Children from households receiving Bolsa Família benefits.  Comparison group: Children from eligible households that did not receive Bolsa Família benefits. | Children  vaccination schedule | No difference or contradictory effects |
| Da Silva & Paes, 2017  [96] | The study dataset included information from the Brazilian Ministry of Social Development (MDS) and the National Health System (SUS), covering Bolsa Família coverage, child mortality rates, prenatal consultations, literacy rates, fertility levels, and access to water and sanitation. | Area, longitudinal ecological study using panel data regression models with fixed effects to assess the association between Bolsa Família participation and child mortality rates. The analysis controlled for social and demographic variables and considered the effect of the Family Health Strategy (ESF) as a key mediator. | Municipalities in the Brazilian semiarid region | 1,133 municipalities | Exposed group: Municipalities with higher Bolsa Família coverage.  Comparison group: Municipalities with lower Bolsa Família coverage, adjusting for socioeconomic factors. | Child mortality rate per 1,000 at municipal level. | Benefit |
| Silva, 2017  [80] | The study was conducted in Brazil, using data from the 2008/2009 Household Budget Survey (Pesquisa de Orçamentos Familiares - POF). | Individual, cross-sectional study using quantile regression models to evaluate the effect of Bolsa Família participation and food price subsidies on children’s Body Mass Index (BMI) and caloric acquisition. Additionally, a household demand system for food consumption was estimated to assess how variations in food prices influenced children's diet. | Children aged 2 to 9, with a per capita household income below 1/2 of the minimum wage. | 6,129 children with resident mothers in the household. | Exposed group: Households receiving Bolsa Família benefits in 2008/2009.  Comparison group: Households eligible but not receiving Bolsa Família benefits | Body Mass Index | No difference or contradictory effects |
| Silvani et al., 2016  [84] | The study was conducted in Porto Alegre, Rio Grande do Sul, Brazil, using primary data collected from 2012 to 2013 in four health units—two Basic Health Units (UBS) and two Family Health Strategy (ESF) units. Food consumption data were obtained via a questionnaire adapted from the SISVAN and VIGITEL national surveys. | Individual, cross-sectional study using descriptive statistics and chi-square tests to compare dietary habits among Bolsa Família beneficiaries and non-beneficiaries. | People aged 18 or more attending a selected Basic Health Unit (UBS) or Family Health Strategy (ESF). | 187 individuals | Exposed group: Adults receiving Bolsa Família benefits.  Comparison group: Adults not receiving Bolsa Família benefits but accessing the same public health units. | Food consumption patterns | Harm |
| Souza et al., 2009  [85] | The study was conducted in the Northeast region of Brazil, using data from the 2002-2003 Household Budget Survey (Pesquisa de Orçamentos Familiares - POF). | Individual, cross-sectional study using logistic multiple regression models to evaluate the effect of conditional cash transfer programs participation on malnutrition and overweight among children and adults. Separate models were estimated for urban and rural areas. | Children aged 6 to 59 months and adults aged 20 to 59 years | 5,267 children (6–59 months); 18,806 adults (20–59 years); Total sample of 18,643 households from the Northeast region. | Exposed group: Individuals from households receiving Bolsa Família or other conditional cash transfers in 2002-2003.  Comparison group: Individuals from households eligible but not receiving Bolsa Família benefits. | Nutritional status | No difference or contradictory effects |
| Souza et al., 2021  [95] | The study was conducted in Brazil, using data from 3,467 municipalities collected annually between 2006 and 2016. The dataset was compiled from publicly available information from the Brazilian Ministry of Health (DATASUS), the Ministry of Social Development (MDS), and the Brazilian Institute of Geography and Statistics (IBGE). | Area, ecological longitudinal study with panel data analysis using generalized linear models (GLM) of the negative binomial type with fixed effects, with and without zero-inflation. Interaction models were applied to assess the combined effects of Bolsa Família coverage and access to improved water, sanitation, and solid waste collection on child morbidity rates. | Children under 5 years old | 38,137 municipal-year observations from 3,467 municipalities over 11 years (2006–2016). | Exposed group: Municipalities with high Bolsa Família coverage and adequate access to water, sanitation, and solid waste collection.  Comparison group: Municipalities with low Bolsa Família coverage or limited environmental sanitation services. | Child morbidity due to diarrhea and malnutrition among children under 5 years old. | No difference or contradictory effects |
| Souza & Heller, 2021  [91] | Systematic review of studies indexed in LILACS, SciELO, and PubMed databases,  published between 2003 (the creation of Bolsa Família) and 2019 | Systematic review of peer-reviewed literature, conducted according to Cochrane Collaboration and PRISMA guidelines and using the following analytical approach: 1)identification of relevant studies; 2) evaluation of study quality using the adapted Downs & Black checklist; 3) extraction of key variables, including intervention type, health outcomes, and statistical measures; 4) synthesis of findings to assess the independent and potential combined effects of Bolsa Família and sanitation on child health. | Children under 5 years old | Articles Initially Identified: 1,658 Studies Included in Final Analysis: 4 | Exposed Group: Children from households receiving Bolsa Família benefits and having access to sanitation services  Comparison Group: Children from households without Bolsa Família benefits and without access to adequate sanitation | Morbidity and mortality due to diarrhea and malnutrition | Benefit |
| Ramos et al, 2021  [92] | Study using data from the 100 Million Brazilian Cohort, which is primarily built from Cadastro Único, Brazil’s Unified Registry for Social Programs. Data linkage was performed using BFP payroll datasets and the Brazilian Mortality Information System (SIM) to analyse child mortality among beneficiaries and non-beneficiaries  in 2006–2015. | Individual level, cross-sectional study using propensity scores and kernel matching and weighted logistic regressions. Propensity score model included the following variables: household density, maternal education, self-reported maternal race/skin color, maternal marital status, maternal parity,  maternal age, region, year of first registration in CadUnico. Variables with more than 10% missing values were not included in the analysis. | Children under 5 years of age whose family enrolled in Cadunico between 2006 and 2015 | 6,309,366 children | Exposed group: Children whose family received a BFP stipend, uninterruptedly, from the first to the fifth year of the child’s life   Comparison group: Children in families that did not receive BFP prior to the child reaching 5 years of age or death, receiving a stipend only after that time point or never receiving a stipend. | Child mortality | Benefit |
| Shei, 2013  [93] | Study using  data from Brazilian Unified Health System database, called DATASUS; the Ministry of Social Development; and the Brazilian Institute of Geography and Statistics.  Analysing data from 2004 to 2009 on the Bolsa Família Program (BFP) and its impact on infant mortality | Area, cross-sectional study using adjusted analysis and pooled, time-series, cross-sectional design. Fixed-effects models used to control for unmeasured time-invariant municipal characteristics (e.g. geography and local cultural practices) and correct for serial correlation of repeated measures. | Infants (0-1 year old) | The study covered municipal-level aggregated data for all municipalities in Brazil over a five-year period. | Exposed group: Municipalities with higher Bolsa Família coverage.  Comparison group: Municipalities with lower Bolsa Família coverage. | Municipal-level all-cause infant mortality rate, post neonatal mortality rate, neonatal mortality rate | Benefit |
| Shei et al, 2014  [94] | The study was conducted in a slum community in a large urban center in Brazil with approximately 14,000 inhabitants. The data was collected through household surveys in 2010, with randomly selected Bolsa Família beneficiaries and non-beneficiaries. | Individual level, cross-sectional study using propensity scores and Propensity score adjustment was used to remove bias associated with differences in the distributions of observed covariates in beneficiary and non-beneficiary groups Observed covariates included characteristics about the child (e.g., age, sex, education), mother (e.g., age, education, work status), and household (e.g., participation in government programs including Bolsa Família, ownership of home, sanitation). | Children  living in a slum community in a large urban center. | 1,266 children | Exposed group: Children from Bolsa Família beneficiary households.  Comparison group: Children from non-beneficiary households. | Children’s health care utilization, check-ups, growth monitoring, psychosocial health, vaccination and diarrhoea | Benefit |
| Sperandio et al, 2017  [86] | The study analysed data from the 2008-2009 Household Budget Survey (Pesquisa de Orçamentos Familiares - POF). | Individual, cross-sectional study using  Propensity Score Matching (PSM) to match beneficiary and non-beneficiary households based on socioeconomic characteristics. The study applied the nearest-neighbor matching algorithm to estimate the program’s impact on underweight, stunting, and overweight prevalence. | Children and adolecesnts aged  5 to 19 years | Northeast region: 6,718 families with had children and adolescents aged 5 to 19 years; of these, 33.0% (N=2,216) were PBF.  In the Southeast region, there were 1,670 such families, and 22.7% (n=379) were PFB. | Exposed group: Children and adolescents from households receiving Bolsa Família benefits.  Comparison group: Children and adolescents from households not receiving Bolsa Família benefits, matched based on socioeconomic characteristics. | Height-for-Age (H/A) and Body Mass Index (BMI)-for-Age (BMI/A) | Benefit |
| Palombo et al., 2024  [83] | Primary data analysis of child health booklet among beneficiaries of the Bolsa Família Program in Salvador-Bahia, Brazil: a cross-sectional study, 2023 | Individual (mothers and children), cross-sectional study using primary data from survey with pre-tested questionnaires and assessment of child health booklets from Family health centers. Used descriptive statistics, chi-square test, and Fisher’s exact test comparing both groups. | Mother-child pairs, majority of mothers Black (94.4%), children aged <6 years in Salvador, Bahia, Brazil in 2023 | 411 mother-child pairs | Exposed group: Mothers receiving Bolsa Familia benefits at the time of the study  Comparison group: Mothers not enrolled in Bolsa Familia Program | Vaccine completeness, child growth and development | Benefit |
| Ferreira et al., 2023  [97] | Study using official vaccination cards and serological tests for measles, mumps, and hepatitis A from Cruzeiro do Sul, Acre, Brazil in 2015-2018 | Individual (children), population-based cohort study using modified poisson regression with robust variance and adjusted for sociodemographic, antenatal care, and nutritional variables | Children, both sexes, aged 2 years | 825 children | Exposed group: Self-reported receipt of Bolsa Familia benefits  Comparison group: Self-reported non-receipt of Bolsa Familia benefits | Vaccination coverage and serological immunity | Harm |
| Santos et al., 2023  [102] | Study using primary data from a validated questionnaire and oral examinations from Family Health Units in Salvador, Bahia, Brazil in 2019 | Individual (children), cross-sectional using poisson regression with robust variance and adjusted for socioeconomic, maternal, and health-related variables | Children, both sexes, aged 6 to 36 months | 535 children | Exposed group: Self-reported receipt of Bolsa Familia benefits  Comparison group: Self-reported non-receipt of Bolsa Familia benefits | Oral health (dental cavities) | Benefit |
| Heckert et al., 2023  [103] | Study using primary data via structured questionnaires and SHP secondary data from Belo Horizonte, Minas Gerais, Brazil in 2021 | Individual (preschool children and their caregivers), cross-sectional descriptive study using descriptive statistics, chi-square test, Fisher’s exact test | Children, median age 5 years, majority male (62.3%) | 61 children | Exposed group: Self-reported receipt of Bolsa Familia benefits  Comparison group: Self-reported non-receipt of Bolsa Familia benefits | Oral health (early childhood caries) | Benefit |
| Csapo et al., 2023  [90] | Study using PNAD, Census, and municipal budget data from Brazil - national in 1997-2010 | Individual (infants) and municipality level, longitudinal ecological study with probit regression and mediation analysis using probit regression, mediation analysis and adjusted for sociodemographic factors, municipal health expenditures, and service availability | Households below the poverty line with infants born within the year prior to the survey | 147632 Households | Exposed group: Households identified through PNAD data as Bolsa Familia beneficiaries  Comparison group: Non-beneficiary households below the poverty line | Infant mortality | Benefit |
| League & Fitz, 2023  [137] | Study using Cadastro Único and climate data from Nationwide (Brazil) in 2001–2009 | Individual (children), natural experiment using exogenous variation using fixed-effects regression and exploiting program rollout variation | Children under 10 years | Varied across analyses | Exposed group: Children in households benefiting from Bolsa Família  Comparison group: Children in similar households but not receiving Bolsa | Child growth and nutrition | Benefit |
| Falcao et al., 2023  [136] | Study using CadÚnico, SINASC, Bolsa Família payroll dataset from National-level study using the 100 Million Brazilian Cohort in 2004-2015 (births from 2012 onward) | Individual level (mother-child dyads), Propensity Score Matching and Weighted Logistic Regression using Propensity Score Matching, Kernel Matching, Weighted Logistic Regression and different subgroup analysis | Live births of mothers registered with CadUnico | 4 277 523 live births | Exposed group: Live births of mothers who had received BFP until delivery (for a minimum of 9 months)  Comparison group: Mothers not receiving Bolsa Família at any time before childbirth | Birth weight indicators (low birth weight, small, and large for gestational age) | Benefit |
| Hilasaca-Mamani et al., 2024  [56] | Study using primary data from na oral health survey, socioeconomic indicators, Bolsa Família status from Municipality of Cajamar, São Paulo, Brazil in 2017 | Individual, ecological study with cluster analysis using cluster analysis, ANOVA, Chi-square and socioeconomic and environmental indicators analyzed with principal component analysis | 1762 schoolchildren from public and private schools | 1762 children | Exposed group: Children from public schools who were Bolsa Família beneficiaries   Comparison group: Non-beneficiary schoolchildren from the same dataset | Oral health and obesity | Harm |
| Ortelan et al., 2024  [135] | Study using CadÚnico, SINASC, Bolsa Família payroll dataset from National-level study using the 100 Million Brazilian Cohort in 2004-2015 (births from 2012 onward) | Individual level (mother-child dyads), Propensity Score Matching and Weighted Logistic Regression using Propensity Score Matching, Kernel Matching, Weighted Logistic Regression and Subgroup analysis by prenatal care quality and Bolsa Família management index | Live births analyzed (65.9% mothers were beneficiaries) | 1031053 live births | Exposed group: Mothers continuously receiving benefits during pregnancy  Comparison group: Mothers not receiving Bolsa Família at any time before childbirth | Preterm births | Benefit |
| Silva et al., 2023  [55] | Study using SISVAN data from primary healthcare service system from Goiânia, Goiás, Brazil in 2013 | Individual level (infants), Bivariate and logistic regression analysis using Bivariate analysis, logistic regression and Comparison of nutritional deviations and breastfeeding practices using ORs and p-values | Children assessed (2.72% BFP beneficiaries) | 4567 children | Exposed group:  children  BFP beneficiaries  Comparison group:  children  non-beneficiaries | Nutritional status and breastfeeding | No difference or contradictory effects |
|  |  |  |  |  |  |  |  |
