# Supplementary material for: Implementation and effects of social protection programs for children, older adults, and people with disabilities in Brazil and Ecuador: A scoping review
Source: PLOS Glob Public Health. 2025 Oct 29;5(10):e0005281. doi: 10.1371/journal.pgph.0005281 (PMC12571297; doi:10.1371/journal.pgph.0005281)
Supplement: S5 Table — (DOCX) [file pgph.0005281.s005.docx]

**S5 Table.** Studies on the effects of the *Bono de Desarollo Humano* on socioeconomic determinants of health or health outcomes (N=15) (Ecuador).

| **Author/Year (et al)** | **Study setting and dataset** | **Study design & methods of analysis** | **Study population** | **Sample size** | **Definition of the exposure and comparison groups** | **Outcome(s)** | **Overall effect direction** |
| --- | --- | --- | --- | --- | --- | --- | --- |
| *Effects on socio-determinants of health* | | | | | | | |
| Ponce & Bedi, 2010  [118] | National study in rural areas using primary data from  Nov/2004 to Feb/2005 | Individual, quantitative/observational study using Regression Discontinuity Design (RDD). The technique of RDD allowed the formation of treatment and control groups (cutoff point 50.65 points on the Selben index), among those who are eligible to receive it. The fuzzy design of the regression discontinuity, which employs instrumental variable techniques, helps mitigate the endogeneity of the treatment variable resulting from contamination (families that did not receive the bonus, even though their score made them eligible, and households that, despite their score not qualifying them for the bonus, ended up receiving it). | School-age children (second and fourth grades) in rural areas | 2,588 | Exposed group: beneficiaries according to Selben Index (<=50,65 points)  Comparison group: non-beneficiaries according to Selben Index i (<=50,65 points) | Cognitive achievement (mathematics and language scores) | No difference |
| Izurieta,2021  [119] | Study using microdata from the national “Ser Bachiller” database and the Associated Factors Survey for the academic cycle 2016-2017 | Individual, quantitative/observational study using Regression Discontinuity Design (RDD). The technique of RDD allowed the formation of treatment and control group (cutoff point 28.2 points on the Social Registry index) The fuzzy design exploits the discontinuity on the probability of being treated by using it as an instrument to explain the treatment status. Fuzzy RDD estimation strategy is a two-stage least squares regression analysis (2SLS). | Adolescents between 17 and 18 years old | 92,367 | Exposed group: beneficiaries according to Social Registry Index (<28.2 points)  Comparison group: non-beneficiaries according to the Social Registry Index (>28.2 points) | Cognitive development (global, mathematics, language, sciences and social studies) and school attendance | Benefit on school attendance |
| Mideros & Gassmann, 2021  [120] | National study using data from the Social Registry (RS) in  three points in time (2002-2003, 2008-2009, 2013-2014) | Quantitative/observational data at the household level using difference-in-difference. By using this targeting rule and exploiting a difference-in-difference (DD) model, it was estimated the intention to treat effect (ITT) in two periods: 2003–2009 and 2009–2014. Given that there is no administrative data to identify actual recipients of the BDH before 2009, it was calculated the effect on those supposed to receive it. Secondly, additional administrative records available for the second period (2009–2014), which indicate actual BDH recipients, allow for estimating average treatment effects (ATE). | Households with the presence of school age children under 18 years old | 413, 043 | Exposed group: beneficiaries' households according to Social registry. The official RS index threshold to receive the BDH was defined for the lowest 40% in 2003 and as a proxy of the consumption poverty line at 50.6 points in 2009 and 36.5 points in 2014.. An additional eligibility condition requires the presence of school age children under 18 years old in the household.  Comparison group: non-beneficiaries households above the cut-off according to the Social Registry | Social mobility | Benefit |
| Guallasamin, 2022  [122] | Study using the national survey“Encuesta de Empleo, desempleo y subempleo (ENEMDU)”  In two points (2010-2012 and 2013-2019) | Quantitative/observational data at the household level using difference-in-differences. The treatment period is defined as the time before the increase in the amount granted by the BDH, that is, before 2013 (𝑡 = 0). On the other hand, the follow-up period corresponds to the values after 2013 (𝑡 = 1). The treatment indicator in the DID framework requires the absence of any intervention at the baseline for any of the groups (𝐷𝑖,𝑡=0 = 0 \| 𝑍𝑖 = 1, 0) and requires that the intervention is positive for the treated group (𝐷𝑖,𝑡=1 = 1 \| 𝑍𝑖 = 1). | Women aged 25 to 55 years and with a maximum total income of $300 per month. The dataset was filtered based on the Multidimensional Poverty Index (MPI) to focus on individuals with an index value greater than 0, meaning multidimensionally poor households. | 2010-2012: 1,964 households 2013-2019: 5,028 households | Exposed group: The treatment group consists of the other households with similar characteristics to control group, who are beneficiaries of the Human Development Bonus transfer.  Comparison group: Control group composed of multidimensional poor households, in which the head of the household is a woman aged between 25 - 55 years, with a maximum total monthly income of $300, and not beneficiaries of the Human Development Bonus transfer. | Multidimensional poverty | Benefit |
| Llerena, 2014  [117] | Study using the national survey “Encuesta de Situación Socioeconómica de Hogares”  in 2011 | Quantitative/observational study at the household level using Regression Discontinuity Design (RDD). To verify the robustness of the results, three intervals were considered in the discontinuity regression. The first interval analyses the variation from 33.59 to 39.59 in the RS Social Registry Index (cutoff point 36.59). That is a variation of 3 points greater than the RS Index (36.59); and 3 points below. The second range implies a range of 5 points at the cut-off point of the Social Registry RS Index. That is, it uses a range of 31.59 to 41.59 of the RS Index. Finally, the results are analysed using a wider range with 7 points at the RS cutoff point (36.59). The range used corresponds to 29.59 to 43.59. Independent variables: age, age squared, head of household, education, if employed and ethnicity with which the person self-identifies. | People between 7 and 29 years old; men and women | 3,593 | Exposed group: beneficiaries at the limit of the BDH cutoff line  Comparison group: No beneficiaries at the limit of the BDH cutoff line | School delay | Benefit in the group aged 13 to 29 years |
| Ponce 2006  [115] | Study using primary data from 4 of 20 provinces in the country: Carchi, Imbabura, Cotopaxi and Tungurahua., all of them located in the highlands. The baseline survey was in June-Aug/2003 and the follow-up survey from Jan-Mar/2005 | Quantitative/observational at household level by combining several methodological approaches: Regression discontinuity (RD), difference-in –difference (DD), and matching estimates. RD used to  identify the impact of the program by simply comparing children pertaining to families scoring just below and above the cutoff point in the Selben index; non-parametric techniques and local linear regression to estimate the limits of equation; 2) DD used to compare the situation of treatment and control groups before starting of the program with the situation after certain period of program application | Children and adolescents aged 6 to 17 years | 2,384 children and 1,463 households | Exposed group: households with at least one child aged from 6 to 15 years old at the time they were surveyed by the Selben - families that score from 47,66 a 50,64 in the Selben index (the cutoff point is 50.65)  Comparison group: families that score from 50.66 to 53.64 in the Selben index (the cutoff point is 50.65) | School enrolment | No difference |
| Seilema & Ramírez , 2016  [123] | Study using the national surveys: “Encuesta de Empleo, desempleo y subempleo (ENEMDU)” and “Encuesta de Condiciones de Vida” from 2007 to 2013 | Quantitative/observational at household level using Cross-sectional analysis The differences between recipients and non-recipients were estimated in absolute and relative values | Older than 10 years old | Not reported | Exposed group: includes all beneficiary profiles  Comparison group: general population not benefited | Poverty, and Gini | Benefit |
| Armas, 2005  [124] | Qualitative diagnosis (field research) - in the cantons of Orellana (Amazon), Machala (Coast) and Riobamba (Highlands) between 2001-2004 | Qualitative at household level using reviews of the program's technical documents and macro-level statistics with the development of a qualitative diagnosis at the local level, through the application of focus groups, workshops with stakeholders and semi-structured interviews | Caregivers of children or people with disabilities, data on children and adolescents, discussion of gender, ethnicity and class | NA | Exposed group: BDH beneficiary women and their specificities such as ethnicity and family composition  Comparison group: NA | Income, inequality and purchase power and includes other categories listed in the options, as well as others related to gender specifics. The categories relate to two populations: firstly, women (also as caregivers) and children and adolescents | No difference or contradictory effects |
| Cecchini et al, 2021  [121] | Study using data from the National Survey of Employment, Unemployment and Underemployment (Encuesta Nacional de Empleo, Desempleo y Subempleo), for 15 Latin American countries, from 2014 to 2017. | Quantitative/observational at the individual level using Cross-sectional. The differences between recipients and non-recipients were estimated in absolute and relative values. | The study investigated the impact of BDH on poverty in the whole population and specific groups. | Not reported | NA | Poverty indicators: incidence (% of people living in poverty), poverty gap (% of people living in poverty weighted by the average distance between their income and the poverty line) and squared poverty gap in the total population and among recipients and non-recipients. | Benefit |
| Schady & Araujo, 2006  [116] | Study using primary data in 4 provinces (Carchi, Imbabura, Cotopaxi, Tungurahua) between 2003 (baseline) and 2005 (follow up) | Quantitative/intervention at household level using RCTs. Linear probability models | Households with children ages 6-17 years. The sample for these calculations is limited to children ages 6-17 at baseline who were re-interviewed in the follow-up survey | 1391 households | Exposed group: One-half of households in the evaluation sample were randomly assigned  to a treatment group that would be eligible for BDH transfers  Comparison group: The other half were assigned to  a control group that would not be eligible for transfers for the first two years | enrolled in school and child work | Benefit |
| Oosterbeek et al, 2008  [114] | Study using primary data in 4 provinces (Carchi, Imbabura, Cotopaxi, Tungurahua) between 2003 (baseline) and 2005 (follow-up) | Quantitative/intervention at the household level using RCTs. Randomized experiment for families around the first quintile of the poverty index and of a regression discontinuity design for families around the second quintile of this index, which is the program’s eligibility threshold | The sample is restricted to children aged 5 to 17 years when they live in households that responded to the follow-up survey.  And with a poverty index between the 13^th^ percentile and the 28^th^ percentile. | 3,004 children in 1,309 families in the experiment (poverty index between 13^th^ and the 28^th^ percentiles), and 2,384 children in 1, 221 households in the RDD study (poverty index between 33^rd^ and the 47^th^ percentile) | Exposed group: "One-half of households in the evaluation sample were randomly assigned  to a treatment group that would be eligible for BDH transfers"  Comparison group: other half were assigned to  a control group that would not be eligible for transfers for the first two years | school enrolment | Benefit |
| ***Effects on health*** | | | | | | | |
| Carranza & Méndez, 2015  [111] | National study using "Encuesta de Condiciones de Vida (ECV)" for 2006 | Quantitative/observational at household level using Regression Discontinuity Design (RDD). The technique of regression discontinuity design allowed the formation of treatment and control groups to estimate the impact of the Human Development Bonus on the exclusive breastfeeding norm in the vicinity of the cutoff point (50.65 points on the Selben index), among those who are eligible to receive it. The fuzzy design of the regression discontinuity, which employs instrumental variable techniques, helps mitigate the endogeneity of the treatment variable resulting from contamination (families that did not receive the bonus, even though their score made them eligible, and households that, despite their score not qualifying them for the bonus, ended up receiving it). | Children <5 years old. l | 1,206 children | Exposed group: households with at least one child aged less than 5 years old at the time of the Living Conditions Survey. Beneficiaries according to Selben index (the cutoff point is 50.65)  Comparison group: Non-beneficiaries' families with a score above the cutoff pointof 50.65 | Exclusive breastfeeding | No difference |
| Fernald & Hidrobo,2011  [112] | Study using data from 378 parishes from six provinces (3 coastal and 3 highland provinces): Baseline (October 2003-March 2004) and follow-up (September 2005- January 2006) | Quantitative/intervention at individual level using RCTs. To estimate the treatment effect of the BDH we took advantage of the randomized roll-out of the program and conducted an intent-to-treat analysis. For the continuous outcome variables (IDHC-B, HAZ, and hemoglobin concentrations), an ordinary least squares regressions were used, and for the dichotomous outcome variable (the indicator representing whether the child had started combining words) probit regressions were used. | Children aged 12-35 months | Baseline (786 children) Follow (1,196 children) | Exposed group: householdswith at least one preschool age child, have no children older than 6 years old, be eligible for the cash transfer program, and not have been recipients of a previous welfare program (Bono Solidario)  Comparison group: households that do not meet the criteria of exposed group | Main outcomes measured were language skills, height-for-age z-score, and haemoglobin concentration. | No difference or contradictory effects |
| Moncayo et al.,2019  [113] | Study using national data from several information systems: National Institute of Statistics and Census (Database of births and deaths, Population Census 2001–2010, hospitalizations) and the National System of Information (Integrated System of Knowledge and Social Statistics of Ecuador and Projections and demographic studies) for 2009-2014 | Quantitative/observational at area level using Longitudinal data. Conditional negative binomial regression models for panel data with fixed-effects specification (counties as units of analysis with observations repeated over time) . The following covariates were used in the analysis: per capita income, illiteracy, percentage of households with inadequate sanitation, total fertility rate, number of physicians per 10,000 residents and bed rate per 1000 residents. | Counties with intermediate and high quality of vital information. | 144 municipalities | Exposed group: BDH gigher levels of coverage on eligible population and county population.  Comparison group: lower levels of coverage. | Mortality of children younger than 5 years, particularly from malnutrition, diarrheal , and lower respiratory tract infections. | Benefit |
| Pozo,2021  [110] | Study using data from the National Survey of Employment, Unemployment and Underemployment (Encuesta Nacional de Empleo, Desempleo y Subempleo) for 2017 | Quantitative/observational at household level using Regression Discontinuity Design (RDD). The technique of regression discontinuity design allowed the formation of treatment and control groups to estimate the impact of the Human Development Bonus on the food insecurity in the vicinity of the cutoff point (34,67906 points on the Social Registry), among those who are eligible to receive it. The fuzzy design of the regression discontinuity, which employs instrumental variable techniques, helps mitigate the endogeneity of the treatment variable resulting from contamination (families that did not receive the bonus, even though their score made them eligible, and households that, despite their score not qualifying them for the bonus, ended up receiving it). | Beneficiaries and non-beneficiaries households divided in various subgroups | 3,892 | Exposed group: beneficiary households according to Social Registry Index (<=34,67906 points)  Comparison group: non-beneficiary households according to Social Registry Index (>34,67906 points) | Household food insecurity | No difference or contradictory effects |
