# Supplementary material for: Implementation and effects of social protection programs for children, older adults, and people with disabilities in Brazil and Ecuador: A scoping review
Source: PLOS Glob Public Health. 2025 Oct 29;5(10):e0005281. doi: 10.1371/journal.pgph.0005281 (PMC12571297; doi:10.1371/journal.pgph.0005281)
Supplement: S6 Table — (DOCX) [file pgph.0005281.s006.docx]

**S6 Table.** Studies on the effects of the Continuous cash benefit on socioeconomic determinants of health or health outcomes (N=5)(Brazil).

| **Author/Year (et al)** | **Study setting and dataset** | **Study design & methods of analysis** | **Study population** | **Sample size** | **Definition of the exposure and comparison groups** | **Outcome(s)** | **Overall effect direction** |
| --- | --- | --- | --- | --- | --- | --- | --- |
| ***Effects on socio-determinants of health*** | | | | | | | |
| Silveira-Neto & Azzoni 2012  [126] | Study using microdata from the National Household Sample Survey (Pesquisa Nacional por Amostra de Domicílios, or PNAD) for years 1995 and 2006. PNAD is a National representative survey. | Ecological, quantitative study using Differences in Differences aggregated by State and Regions in Brazil. | Authors used microdata of income from PNAD but aggregated into States and regions of Brazil. Total income was split into two broad categories: labour-related and non-labor-related. They consider five sources of non-labour-related income: retirement payments and pensions, property rents and other income, capital income (interests and dividends), and two government social programs—Bolsa Familia (BF) and BPC. | 27 States, 5 regions | Exposed group: Higher proportion of income due to BPC  Comparison group: Lower proportion of income due to BPC | Gini Index (Inequality) (continuous) | benefit |
| Kassouf & Oliveira 2012  [125] | Study using microdata from the National Household Sample Survey (Pesquisa Nacional por Amostra de Domicílios, or PNAD) for years 2001 and 2008. PNAD is a National representative survey. | Individual, quantitative study using Regression Discontinuity Design (RDD) exploring the change in eligibility from 67 up to 2003 to 65 after that and using age as the cut-off point, exploring the difference in the outcome variables around the cutoff point. Differences in difference and propensity score matching were also used as secondary methods of analysis. | Authors used microdata from PNAD of individuals around the threshold of age to receive BPC as well as their co-residents. | Sample sizes depended on the outcomes: 1. 7512 2. 7512 3. 1768 4. 9969 5. 5069 6. 3285 7. 3285 | Exposed group: Above the age cut-off point (age>=66 and age>67)  Comparison group: Below the age cut-off point (age>=66 and age>67) | 1. Labor Force Participation (month) 2. Labor Force Participation (week) 3. Weekly worked hours 4. Co-residents 18-49y labour force participation (month) 5. Co-residents 18-29y labour force participation (month) 6. Child Labour (10-15y) 7. School attendance (10-15y) | benefit |
| ***Effects on health*** | | | | | | | |
| Cintra et al 2017  [127] | Study using national representative survey data from PNAD (Pesquisa Nacional por Amostra de Domicílios) 2008. | Individual, cross-sectional study with descriptive statistics of all eligible people obtained from PNAD 2008. The authors compared the healthy life expectancy calculated by the Sullivan method (combination of age and self-perception of health in terms of very good to very bad). They calculated the estimated years by age group in three groups: 1. people aged >= 65y who possibly received BPC; 2. People who possibly did not receive BPC but were eligible in terms of related age and income from work; 3. People with a household per capita income between 1/4 and 1 minimum wage (not eligible for BPC). | The final sample of the three groups was comprised of 593,954 people aged >= 65y who possibly received BPC, 315,530 people aged >= 65y with a per capita income of up to ¼ minimum wage, but who did not receive BPC, and 2,207,895 elderly people with a household per capita income of up to 1 minimum wage | 3,117,379 | Exposed group:  Group 1 - People aged >= 65y who possibly received BPC (i.e., with a monthly per capita income from work of up to ¼ minimum wage and with benefits of exactly 415 reais which were the value of BPC benefit)  Comparison groups:  Group 2- People with a household per capita income between 1/4 and 1 minimum wage (not eligible for BPC).  Group 3 - Elderly people eligible for BPC but not receiving the benefit for unknown reasons | Healthy life expectancy (assessed using self-reported health status: “very good” e “good” = good self-perception of health, and “regular”, “bad” e “very bad” = bad self-perception of health) | benefit |
| Rosales et al 2023  [128] | Study using data from the 2017–2018 Consumer Expenditure Survey (POF) conducted in a sample of households in Brazil. | Individual, quantitative study using a fuzzy regression discontinuity design (RDD) using age as a forcing variable (eligibility) and further adjusted for socioeconomic variables. Estimates were presented for different bandwidths and with and without adjustments. | Individuals aged 57 to 72 years living in households randomly surveyed by POF, which include individuals of all income levels. |  | Exposed group: older people in households that are benefited from BPC at the time of the survey  Comparison group: older people in households that are NOT benefited from BPC at the time of the survey | Obesity, undernutrition, and food security and food insecurity (mild, moderate or severe) | Benefit (food security) and no difference (others) |
| Aransiola et al 2024  [129] | Study using municipal-level aggregated data from several data sources over time with yearly data from 2004 to 2019. | Ecological, quantitative study using Differences in Differences with Propensity score matching (PSM) by socioeconomic and demographic municipal characteristics, as well as adjusted by other programmes (Bolsa Familia Programme and Family Health strategy) at the municipality level. | Municipalities with adequate vital statistics. The study population was stratified by age, and results were presented for individuals aged < 5 years, 5-29 years, 30-69 years and ≥70 years. The study used data from multiple sources, including census, surveys and from the Ministry of Health and the Ministry of Social Development. | 2548 | Exposed group: Higher levels of municipality BPC coverage in relationship to the total population divided into consolidated coverage (66–100%) and Intermediate coverage (33–66%)  Comparison group: Lower levels of BPC coverage in relationship to the total population | All-cause mortality ≥70 years  Hospitalizations for all causes and all age groups (data not shown only for older people) | benefit |
