# Supplementary material for: Implementation and effects of social protection programs for children, older adults, and people with disabilities in Brazil and Ecuador: A scoping review
Source: PLOS Glob Public Health. 2025 Oct 29;5(10):e0005281. doi: 10.1371/journal.pgph.0005281 (PMC12571297; doi:10.1371/journal.pgph.0005281)
Supplement: S2 File — (DOCX) [file pgph.0005281.s008.docx]

**Membership of the Unit on the Social and Environmental Determinants of Health Inequalities (SEDHI)**

**Format: Surname, First name, Middle name, Department, Institution, City, Country (e-mail).**

**Alvarez, Pablo, Universidad Internacional del Ecuador, Ecuador (**[**palvarez@uide.edu.ec**](mailto:palvarez@uide.edu.ec)**); Almeida, Cristina, Fiocruz, Brazil (**[**cristiana.almeida@fiocruz.br**](mailto:cristiana.almeida@fiocruz.br)**); Aldaz Barreno, Cristina Elizabeth, Universidad Internacional del Ecuador, Ecuador (**[**craldazba@uide.edu.ec**](mailto:craldazba@uide.edu.ec)**); Andrade, Roberto F.S., Physics Institute and Centre for Data and Knowledge Integration for Health-CIDACS, Federal University of Bahia/Fiocruz, Salvador, Brazil (**[**randrade@ufba.br**](mailto:randrade@ufba.br)**); Andrade Ortiz, Diego Oswaldo,  Universidad Internacional del Ecuador, Quito , Ecuador (**[**diandradeor@uide.edu.ec**](mailto:diandradeor@uide.edu.ec)**); Anjos, EF, National School of Public Health (ENSP), Centre for Data and Knowledge Integration for Health (CIDACS), Fiocruz, Brazil (**[**eduarda.anjos@fiocruz.br**](mailto:eduarda.anjos@fiocruz.br)**); Araújo da Cruz Casais e Silva, Luiz Gustavo, Fiocruz, Brazil (**[**lgcasais3@gmail.com**](mailto:lgcasais3@gmail.com)**); Arcos Garcia, Pamela Alejandra, Universidad Internacional del Ecuador, Ecuador (**[**parcos@uide.edu.ec**](mailto:parcos@uide.edu.ec)**); Barreto , Mauricio , Centre for Data and Knowledge Integration for Health-CIDACS, Fiocruz, Salvador, Brazil (**[**mauricio.barreto@fiocruz.br**](mailto:mauricio.barreto@fiocruz.br)**); Bernal RTI, Programa de Pós-graduação em Saúde Pública, Universidade Federal de Minas Gerais, Belo Horizonte, Brazil (**[**reginabernal@terra.com.br**](mailto:reginabernal@terra.com.br)**); Borbor Cordova, Mercy Julia, Escuela Superior Politécnica del Litoral, Ecuador (**[**meborbor@espol.edu.ec**](mailto:meborbor@espol.edu.ec)**); Brickley , Elizabeth, London School of Hygiene & Tropical Medicine, UK (**[**elizabeth.brickley@lshtm.ac.uk**](mailto:elizabeth.brickley@lshtm.ac.uk)**); Burke, Claire, University of Glasgow, Glasgow, UK (**[**claire.burke@glasgow.ac.uk**](mailto:claire.burke@glasgow.ac.uk)**); Butler, Brenda, University of Glasgow, Glasgow, UK (**[**brenda.butler@glasgow.ac.uk**](mailto:brenda.butler@glasgow.ac.uk)**); Calderon Huachi, Nina Micaela, University of Glasgow, Glasgow, UK (**[**2947863C@student.gla.ac.uk**](mailto:2947863C@student.gla.ac.uk)**); Campbell, Desmond D, School of Health and Wellbeing, University of Glasgow, Glasgow, UK (**[**desmond.campbell@glasgow.ac.uk**](mailto:desmond.campbell@glasgow.ac.uk)**); Campbell, Mhairi, University of Glasgow, UK (**[**mhairi.campbell@glasgow.ac.uk**](mailto:mhairi.campbell@glasgow.ac.uk)**); Carrero, Roberto, Fiocruz, Brazil (**[**roberto@perezcarreiro.org**](mailto:roberto@perezcarreiro.org)**); Cardoso LSM, Programa de Pós-graduação em Saúde Pública, Universidade Federal de Minas Gerais, Belo Horizonte, Brazil (**[**laissmcardoso@gmail.com**](mailto:laissmcardoso@gmail.com)**); Cerezo, Jose L, University of Glasgow, Glasgow, UK (**[**jose-luis.cerezo-Zambudio@glasgow.ac.uk**](mailto:jose-luis.cerezo-Zambudio@glasgow.ac.uk)**); Chis Ster, Irina, St George’s University of London, London, UK (**[**ichisste@sgul.ac.uk**](mailto:ichisste@sgul.ac.uk)**); Constatine Tigua, Annabel del Rocio, Escuela Superior Politécnica del Litoral, , Ecuador (anndcons@espol.edu.ec ); Cooper, Philip J, Institute of Infection and Immunity, St George’s University of London, London, UK (**[**pcooper@sgul.ac.uk**](mailto:pcooper@sgul.ac.uk)**); Costa, Marianne, Fiocruz, Salvador, Brazil (**[**marianne.lage@fiocruz.br**](mailto:marianne.lage@fiocruz.br)**); Correa Matta, Gustavo, Fiocruz, Brazil (**[**gcmatta@gmail.com**](mailto:gcmatta@gmail.com)**); Craig , Peter, University of Glasgow, Glasgow, UK (**[**peter.craig@glasgow.ac.uk**](mailto:peter.craig@glasgow.ac.uk)**); Cruz, Enny Paixao, London School of Hygiene & Tropical Medicine, (**[**enny.cruz@lshtm.ac.uk**](mailto:enny.cruz@lshtm.ac.uk)**); de Araujo Almeida, Bethânia, Fiocruz, Brazil (**[**bethania.almeida@fiocruz.br**](mailto:bethania.almeida@fiocruz.br)**); de Carvalho Neto, Edgar Marcelino, Fiocruz, , Brazil (**[**edgar.neto@fiocruz.br**](mailto:edgar.neto@fiocruz.br)**); de Jesus Neves, Felix, Fiocruz, Brazil (**[**felixnevesjr@hotmail.com**](mailto:felixnevesjr@hotmail.com)**); de Oliveira Ramos, Dandara, Fiocruz, , Brazil (**[**dandara.ramos@ufba.br**](mailto:dandara.ramos@ufba.br)**); de Souza Dias, Francine, Fiocruz, Brazil (**[**francine.dias@fiocruz.br**](mailto:francine.dias@fiocruz.br)**); Dundas, Ruth, MRC/CSO Social and Public Health Sciences Unit, University of Glasgow, Glasgow, UK (**[**ruth.dundas@glasgow.ac.uk**](mailto:ruth.dundas@glasgow.ac.uk)**); Einloft, ABN, Centre for Data and Knowledge Integration for Health-CIDACS, Fiocruz, Brazil (**[**ariadne.einloft@fiocruz.br**](mailto:ariadne.einloft@fiocruz.br)**); Emanuel da Silva, Lucas, Federal University of Bahia, Brazil (**[**lucasemanuel@ufba.br**](mailto:lucasemanuel@ufba.br)**); Fonseca, A. A., Fiocruz, Salvador, Brazil (**[**adalton.anjos@gmail.com**](mailto:adalton.anjos@gmail.com)**); Fernandes Silva Andrade, Roberto, Fiocruz, Brazil (**[**randrade@ufba.br**](mailto:randrade@ufba.br)**); Fiaccone Leovigildo, Rosemeire, Federal University of Bahia, Brazil (**[**rose.fiaccone@gmail.com**](mailto:rose.fiaccone@gmail.com)**); Flores-Quispe, María del Pilar, Fiocruz, Brazil (**[**maria.quispe@fiocruz.br**](mailto:maria.quispe@fiocruz.br)**); Franco Sansigolo Kerr, Ligia, Federal University of Ceará, Fortaleza, CE, Brazil (**[**ligiakerr@gmail.com**](mailto:ligiakerr@gmail.com)**); Ferreira dos Santos, Gervásio, Federal University of Bahia, Brazil (**[**gervasios@ufba.br**](mailto:gervasios@ufba.br)**); Goes, Emmanuelle, Fiocruz, Brazil (**[**emanuellegoes@gmail.com**](mailto:emanuellegoes@gmail.com)**); Gonzaga, Marcos, University of Rio Grande do Norte (UFRN), Brazil (**[**marcos.gonzaga@ufrn.br**](mailto:marcos.gonzaga@ufrn.br)**); Gualan, Monsermin, Universidad Internacional del Ecuador, Ecuador (**[**mogualanch@uide.edu.ec**](mailto:mogualanch@uide.edu.ec)**); Guimarães, JMN, Center for Data and Knowledge Integration for Health-CIDACS, Fiocruz, Salvador, Brazil (**[**joannaguimaraes@hotmail.com**](mailto:joannaguimaraes@hotmail.com)**); Hargreaves, Sally, Institute for Infection and Immunity, St George’s University of London, London, UK (**[**s.hargreaves@sgul.ac.uk**](mailto:s.hargreaves@sgul.ac.uk)**); Harron, Katie , Population, Policy and Practice, Great Ormond Street Institute of Child Health, University College London, London, UK (**[**k.harron@ucl.ac.uk**](mailto:k.harron@ucl.ac.uk)**); Herculano de Morais, Évelin Angélica, Universidad Federal de Minas Gerais, Brazil (**[**moraisevelinah@gmail.com**](mailto:moraisevelinah@gmail.com)**); Ichihara, Maria Yury, Cidacs, Fiocruz, Salvador, BA, Brazil (**[**maria.yury@fiocruz.br**](mailto:maria.yury@fiocruz.br)**); Katikireddi, Vittal, University of Glasgow, Glasgow, UK (**[**vittal.katikireddi@glasgow.ac.uk**](mailto:vittal.katikireddi@glasgow.ac.uk)**); Kendall, Carl, Graduate Program in Public Health, Federal University of Ceará , Fortaleza CE, Brazil (**[**carl.kendall@gmail.com**](mailto:carl.kendall@gmail.com)**); Leyland, AH, MRC/CSO Social and Public Health Sciences Unit, University of Glasgow, Glasgow, UK (**[**alastair.leyland@glasgow.ac.uk**](mailto:alastair.leyland@glasgow.ac.uk)**); Lilford, RJ, Institute of Applied Health, University of Birmingham, Birmingham, UK (**[**r.j.lilford@bham.ac.uk**](mailto:r.j.lilford@bham.ac.uk)**); Llangari Arizo, Luz Marina, Universidad Internacional del Ecuador, Ecuador (**[**lullangari@uide.edu.ec**](mailto:lullangari@uide.edu.ec)**); Lopes, D., Center for Data and Knowledge Integration for Health-CIDACS, Fiocruz, Salvador, BA, Brazil (davidlopes.educacao@gmail.com); Lowe, Rachel, London School of Hygiene & Tropical Medicine, UK (**[**rachel.lowe@lshtm.ac.uk**](mailto:rachel.lowe@lshtm.ac.uk)**); Lugo, Daniela, Universidad Internacional del Ecuador, Ecuador (**[**dalugoro@uide.edu.ec**](mailto:dalugoro@uide.edu.ec)**); Macdonald, Sara, University of Glasgow, UK (**[**sara.macdonald@glasgow.ac.uk**](mailto:sara.macdonald@glasgow.ac.uk)**); Malta DC, Departamento de Enfermagem Materno-Infantil e Saúde Pública, Universidade Federal de Minas Gerais, Belo Horizonte, Brazil (**[**dcmalta@uol.com.br**](mailto:dcmalta@uol.com.br)**); Mendes Pereira, Adelyne, Fiocruz, Brazil (**[**adelyne.mendes@fiocruz.br**](mailto:adelyne.mendes@fiocruz.br)**); Mendoza Ruiz, Adriana, Fiocruz, , Brazil (**[**adriana.mendoza@fiocruz.br**](mailto:adriana.mendoza@fiocruz.br)**); Moncayo Benalcazar, Ana Lucía, Centre for Research on Health in Latin America, (**[**amoncayo708@puce.edu.ec**](mailto:amoncayo708@puce.edu.ec)**); Moraes Pimenta, Denise, Fiocruz, Sao Paolo, Brazil (**[**denise.mpimenta@fiocruz.br**](mailto:denise.mpimenta@fiocruz.br)**); Navarrete Chavez, Grace, Universidad Internacional del Ecuador, Quito/Glasgow, UK (**[**2947619N@student.gla.ac.uk**](mailto:2947619N@student.gla.ac.uk)**); Nascimento, Erica Miranda, Fiotec, Brazil (**[**ericanascimento@fiotec.fiocruz.br**](mailto:ericanascimento@fiotec.fiocruz.br)**); Nunes de Carvalho, Rumão Batista, Brazil (rumao.carvalho@fiocruz.br); Oliveira Junker, Sara Caroline, Fiotec, Brazil (**[**sarajunker@fiotec.fiocruz.br**](mailto:sarajunker@fiotec.fiocruz.br)**); Oliveira Lima Sena, Samila, Fiocruz, Brazil (**[**samila.sena@fiocruz.br**](mailto:samila.sena@fiocruz.br)**); Jonathan R Olsen, MRC/CSO Social and Public Health Sciences Unit, University of Glasgow, Glasgow, UK (**[**jonathan.olsen@glasgow.ac.uk**](mailto:jonathan.olsen@glasgow.ac.uk)**); Oliveira, Suelen, National School of Public Health (ENSP), Fiocruz , Rio de Janeiro, Brazil  (**[**suelen.coliveira@fiocruz.br**](mailto:suelen.coliveira@fiocruz.br)**); Ortelan, Naiá, Fiocruz, Brazil (**[**naia.ortelan@fiocruz.br**](mailto:naia.ortelan@fiocruz.br)**); Pinto Junior, EP, Center for Data and Knowledge Integration for Health-CIDACS, Fiocruz, Salvador, Brazil (elzo.junior@fiocruz.br); Pita, Robespierre, Fiocruz, Brazil (**[**pierre.pita@gmail.com**](mailto:pierre.pita@gmail.com)**); Julia M Pescarini, Centro de Integracao de Datos e Cohecimentos para Saude (CIDACS), Instituto Gongalo Moniz, Fiocruz, Salvador, Brazil (**[**Julia.Pescarini1@lshtm.ac.uk**](mailto:Julia.Pescarini1@lshtm.ac.uk)**); Ramos DO, Institute of Collective Health, Federal University of Bahia, Salvador-Bahia, Brazil (**[**dandara.ramos@ufba.br**](mailto:dandara.ramos@ufba.br)**); Ribeira, Rita, Federal University of Bahia, Brazil (**[**ritaribeiroufba@gmail.com**](mailto:ritaribeiroufba@gmail.com)**); Rodriguez Alvarado, Rodrigo Alejandro, Universidad Internacional del Ecuador, Ecuador (rorodriguezal@uide.edu.ec); Batul Rojeab Bravo, St George’s University of London, London, UK (**[**m2307408@sgul.ac.uk**](mailto:m2307408@sgul.ac.uk)**); Romero Sandoval, Natalia Cristina, Universidad Internacional del Ecuador, Quito, Ecuador (**[**nromero@uide.edu.ec**](mailto:nromero@uide.edu.ec)**); Revoredo, Fernanda, Fiocruz, Salvador, Brazil (**[**fernanda.revoredo@fiocruz.br**](mailto:fernanda.revoredo@fiocruz.br)**); Ruano, Maria Alejandra, Facultad de Ciencias Sociales y Humanísticas, Escuela Superior Politecnica del Litoral, Guayaquil, Ecuador (**[**maruano@espol.edu.ec**](mailto:maruano@espol.edu.ec)**); Rui Chng, Nai, University of Glasgow, UK (**[**NaiRui.Chng@glasgow.ac.uk**](mailto:NaiRui.Chng@glasgow.ac.uk)**); Ruiz, Ronal, Universidad Internacional del Ecuador, Ecuador (roruiz@uide.edu.ec); Sande, Raphael, Fiocruz, Brazil (**[**raphael.sande@fiocruz.br**](mailto:raphael.sande@fiocruz.br)**); Santos de Jesus, T., Centre for Data and Knowledge Integration for Health-CIDACS, Fiocruz, Salvador, Brazil (**[**talita.jesus@fiocruz.br**](mailto:talita.jesus@fiocruz.br)**); Santucci, Paula M, Centre for Data and Knowledge Integration for Health-CIDACS, Fiocruz, Salvador, BA, Brazil (**[**paulamsantucci@gmail.com**](mailto:paulamsantucci@gmail.com)**); Scandrett, Katie Ellen, University of Birmingham, UK (**[**k.e.scandrett@bham.ac.uk**](mailto:k.e.scandrett@bham.ac.uk)**); SEBASTIAO, M. R., Cidacs, Fiocruz, Brazil (**[**mariana.sebastiao@fiocruz.br**](mailto:mariana.sebastiao@fiocruz.br)**); Sebastiao, Mariana, Fiocruz, Brazil (**[**mariana.sebastiao@fiocruz.br**](mailto:mariana.sebastiao@fiocruz.br)**); Silva de Jesus, Viviane, Fiocruz, Brazil (**[**vivi_humaniza@hotmail.com**](mailto:vivi_humaniza@hotmail.com)**); Shimonovich, Michal, University of Glasgow, Glasgow, UK (**[**michal.shimonovich@glasgow.ac.uk**](mailto:michal.shimonovich@glasgow.ac.uk)**); Souza , Maira Lima, Fiocruz, Brazil (**[**maira.souza@fiocruz.br**](mailto:maira.souza@fiocruz.br)**); Sousa Filho, J.F., Fiocruz, Brazil (**[**jose.ffilho@fiocruz.br**](mailto:jose.ffilho@fiocruz.br)**); Taurihno Lima, Raiza, Fiocruz, Brazil (**[**raizatourinho@gmail.com**](mailto:raizatourinho@gmail.com)**); Tavares de Souza Junior, Cleônidas, Fiocruz, Brazil (cleonidas@gmail.com ); Teles , Carlos, Fiocruz, Brazil (**[**carlosateles@yahoo.com.br**](mailto:carlosateles@yahoo.com.br)**); Vera Alcivar, David Gonzalo, Universidad Internacional del Ecuador, Ecuador (**[**daveraal@uide.edu.ec**](mailto:daveraal@uide.edu.ec)**); Vieira Machado, Cristiani, Fiocruz, Brazil (**[**cristiani.machado@fiocruz.br**](mailto:cristiani.machado@fiocruz.br)**); Wells, Valerie, University of Glasgow, UK (**[**valerie.wells@glasgow.ac.uk**](mailto:valerie.wells@glasgow.ac.uk)**); Zurita, Daniel, Universidad Internacional del Ecuador, Ecuador (dzurita@uide.edu.ec);**
